# Analysis of Aldehyde Dehydrogenase 2 as a Prognostic Marker Associated with Immune Cell infiltration and Chemotherapy Efficacy in Head and Neck Squamous Cell Carcinoma

**DOI:** 10.7150/jca.85098

**Published:** 2023-06-12

**Authors:** Yu-Hsuan Lin, Yi-Fang Yang, Jia-Bin Liao, Ting-Shou Chang, Uyanahewa Gamage Shashini Janesha, Yow-Ling Shiue

**Affiliations:** 1 Institute of Biomedical Sciences, National Sun Yat-Sen University, Kaohsiung 804, Taiwan.; 2 Department of Otolaryngology, Head and Neck Surgery, Kaohsiung Veterans General Hospital, Kaohsiung 813, Taiwan.; 3 School of Medicine, National Yang Ming Chiao Tung University, Taipei 112, Taiwan.; 4 School of Medicine, Chung Shan Medical University, Taichung 402, Taiwan.; 5 Department of Medical Education and Research, Kaohsiung Veterans General Hospital, Kaohsiung 813, Taiwan.; 6 Department of Pathology and Laboratory Medicine, Kaohsiung Veterans General Hospital, Kaohsiung 813, Taiwan.; 7 Institute of Precision Medicine, National Sun Yat-sen University, Kaohsiung, Taiwan, Kaohsiung 804, Taiwan.

**Keywords:** Aldehyde Dehydrogenase 2, head and neck squamous cell carcinoma, human papillomavirus, tumor microenvironment, 5-fluorouracil

## Abstract

**Background:** Previous investigations have demonstrated the role of *Aldehyde Dehydrogenase 2* (*ALDH2*) levels in the cancer initiation and progression, prognosis, and treatment response in kinds of malignancies. However, its significance in the head and neck squamous cell carcinoma (HNSC) by different human papillomavirus (HPV) statuses remains unclear.

**Methods:** We conducted an in-depth analysis of *ALDH2* in HNSC using various bioinformatics tools, investigating its expression, alteration, differential levels, prognostic significance, molecular interactions, immune characteristics, and conducting experimental validation through immunohistochemistry (IHC) arrays and Western blot to compare expression levels between tumor and normal tissues, analyze the associations with clinicopathological features, and investigate its responses to chemotherapies.

**Results:** *ALDH2* levels are downregulated in HNSC tissues and associated with higher American Joint Committee on Cancer (AJCC) T classification and worse overall survival in HPV-unrelated HNSC, yet not in HPV-related HNSC. *ALDH2* is positively regulated by copy-number variation and negatively regulated by DNA methylation. The association of *ALDH2* with prognosis may be due to its interaction with *ALDH6A1*, and its co-expressed genes are predictive biomarkers of HNSC. We also found high *ALDH2* levels in bulk tumors are associated with increased immune surveillance cells, such as naïve B cells and M1 macrophages in HPV-unrelated HNSC. IHC and western blot showed that ALDH2 is downregulated in the oral cavity, hypopharyngeal cancers, and well-differentiated carcinoma. *In vitro*, low *ALDH2* levels showed reduced response to 5-fluorouracil in HNSC-derived cell lines.

**Conclusion:** Our analyses revealed the genetic and cellular targets and drug response of ALDH2 in HNSC. We also found *ALDH2* is involved in regulating the immune response of the tumor microenvironment, and high levels of *ALDH2* in bulk HNSC may enhance antitumor immunity, which could improve prognosis. These findings suggest that *ALDH2* could be a potential biomarker in improving risk stratification and tailoring treatment strategies in HNSC patients, especially in the HPV-unrelated subgroup.

## Introduction

Head and neck squamous cell carcinoma (HNSC) is prevalent cancer with increasing incidence globally, attributed to various risk factors, including tobacco, alcohol, betel nut use, and human papillomavirus (HPV) infection 1. HPV-related and HPV-unrelated HNSCs display different biological behaviors, with HPV-related HNSC historically conferring better prognoses and less therapeutic resistance 1. A comprehensive genomic analysis of 120 locoregionally advanced HNSC patients showed that the expression levels of numerous transcripts predominantly altered in HPV-related and HPV-unrelated HNSCs 2. Specifically, HPV-unrelated HNSC is characterized by co-amplifications of specific genes on chromosomes 11q13 and 11q22 and recurrent somatic mutations of *TP53*, *CKDN2A*, *FAT1*, and *AJUBA* 2. On the other hand, the features of the HPV-related subset are focal amplification of *PIK3CA*, *E2F*1, and recurrent deletion of *TRAF3* 2. However, the altered transcripts are not therapeutically relevant. Thus, there is a need to identify clinically actionable molecules for risk stratification and improvement of prognoses, especially the HPV-unrelated HNSC.

ALDH2 is a mitochondrial isoenzyme found in all tissues that comprise 13 exons and 517 amino acids 3,4. Its primary function is detoxifying endogenous and exogenous aldehydic products and maintaining redox balance 3,4. The *ALDH2* single nucleotide polymorphism (SNP) rs671, a G to A transition at position 42421, produced ALDH2K protein from the ALDH2*2 allele, which reduces enzyme activity with a shorter half-life, rendering cells more susceptible to damage from oxidative reactions 3,4. The mutation associates with the initiation and progression of stress-related disorders and cancer through various mechanisms 3,4. In contrast, research suggests that *ALDH2* levels, rather than *ALDH2* variants, participate in the regulation of carcinogenesis and cancer behaviors through genetic instability 5-7, self-renewal of cancer stem cells 8, and tumor immune microenvironment 9-11. Recent research showed that *ALDH2* repression contributes to liver cancer development by inhibiting the expression of HBV peptide-MHC class I complexes and reducing the activation of cytotoxic T lymphocytes 9. A*LDH2* knockout in *Fanconi Anemia Complementation Group D2 (FANCD2*^-/-^) mice leads to deletion and rearrangements of stem cells and increased micronuclei spillage of peripheral blood mononuclear cells, resulting in leukemia development 6. *ALDH2* levels are significantly downregulated in liver cancer tissues than in normal tissues, and low tumor *ALDH2* levels are associated with migration-related traits and poor survival 12. Downregulation of *ALDH2* has been demonstrated to improve the efficacy of anthracyclines in renal cell carcinoma via *von Hippel-Lindau* (*VHL*) deficiency 13 and to sensitize lung cancers to paclitaxel by reducing cancer stemness 14. However, the role of ALDH2 levels in head and neck squamous cell carcinoma is yet to be fully understood.

Given the distinct genomic features based on HPV status and the low incidences of *ALDH2* mutation (2/523, 0.4 %) in the HNSC/TCGA cohort, we used a multi-omic strategy to characterize the effects of *ALDH2* on the biological behaviors, phenotypes, and prognoses of HNSC by HPV status. This study also explored the potential mechanisms of *ALDH2* by utilizing molecule interaction prediction, gene set enrichment, and genetic signature association analyses with tumor immune microenvironments. We further investigated the treatment responses to systemic agents based on *ALDH2* levels.

## Material and Methods

### The Cancer Genome Atlas (TCGA) cohort's preparation

#### Patients and datasets

We downloaded the clinical information and RNA sequencing (RNA-Seq) data of 523 HNSC and another 44 healthy samples from cBioPortal (https://www.cbioportal.org/) and UCSC Xena platform (https://xena.ucsc.edu/) up to Feb 2023. RNA-Seq measured the gene expression and showed the Expectation-Maximization (RSEM). cBioPortal provides information, including mutation count, microsatellite instability scores, tumor mutation burden, age of diagnosis, HPV status, histology grade, disease status, intervals from events, and vital status. The UCSC Xena browser distributes the data of anatomic subsite, AJCC T- and AJCC- N classifications, the presence of perineural invasion and lymphovascular invasion, surgical margin, the performance of lymph node, total positive lymph node yield, and the substance use. The substance use includes positive or negative users, based on whether the patients had active or ever cigarette or alcohol use. Five patients with an initial diagnosis of distant metastasis and 28 patients without complete data were excluded. A total of 490 HNSC patients were subjected to further analysis.

#### Clinical samples and HNSC tissues microarrays

Human cancer and paired normal tissues of 3 patients with hypopharyngeal cancer who underwent tumor biopsy at the Kaohsiung Veterans General Hospital between 2022 Jan and 2022 Jun were collected. These patients had provided written informed consent, and the study received approval from the Institutional Review Board of Kaohsiung Veterans General Hospital (IRB: KSVGH21-CT6-16). The tissue microarrays (TMAs) (HN721, HN802c) consisting of 133 patients and 19 unpaired healthy tissues were obtained from Biommax (Rockville, MD, USA). The ALDH2 antibody (GTX101429, Genetex) was used for immunohistochemistry (IHC) (1:500). A pathologist evaluated the TMAs to exclude the microarray without good quality, and the ALDH2 IHC was then quantified using the HistoQuest software (version 7.1 Nuclear Segmentation using deep learning) for further analysis.

#### Gene alteration analysis

The cBioPortal demonstrated the types and frequencies of *ALDH2* alterations in TCGA cohorts, the altered transcripts with *ALDH2* alteration, and the information on the copy number alteration and DNA methylation on *ALDH2* mRNA levels. The 'Plots' module of cBioPortal estimated the associations between the *ALDH2* level and tumor mutation burden and microsatellite instability. The Spearman and Pearson correlation tests analyzed the associations, and a *p*-value < 0.05 indicates statistical significance.

#### Gene, protein expression, and survival analyses

TIMER 2.0 (http://timer.cistrome.org/) and UALCAN (http://ualcan.path.uab.edu/index.html) explored the *ALDH2* levels (Transcript Per Million, TPM) in bulk tumor and normal tissues from TCGA cohorts. We then evaluate the effects of clinical variables, HPV status, and gene mutation on *ALDH2* levels. TNMplot (https://tnmplot.com/analysis/) analyzed the differential expression in the GeneChips and RNA-seq. The ALDH2 protein levels were accessed using UALCAN, which sorted the data from the Clinical Proteomic Tumor Analysis Consortium (CPTAC) prediction. The correlations between mRNA levels of transcripts were analyzed using TIMER 2.0 to generate correlation coefficients and estimate the *p*-value. The correlations between clinicopathologic variables and *ALDH2* mRNA or protein levels were performed using the SPSS software. Parametric tests, such as the Student *t*-test and Chi-square test, and nonparametric tests, such as the Mann-Whitney U test and Fisher's exact test, were used to analyze the data. Kaplan-Meier method analyzed the prognostic significance of *ALDH2* level on overall and disease-specific survival rates. The log-rank test compared the survival rates between groups, and a significance threshold is *p*-value < 0.05.

#### Molecular interaction network and enrichment analyses

The STRING (https://www.string-db.org/) and GeneMANIA (https://genemania.org/) databases generate protein and gene networks to identify the significant molecules interacting with ALDH2. STRING focuses on the strength and sources of protein-protein interactions and functional enrichment analysis, while GeneMANIA provides information on genetic interactions, gene functions, and related pathways. The LinkedOmics Database (http://linkedomics.org/admin.php) identified *ALDH2*-coexpressed transcripts in 517 patients (520 microarrays) from the TCGA-HNSC cohort [*P* < 0.01, false discovery rate (FDR) < 0.01]. The prognostic significances of the top 30 positively and negatively co-expressed transcripts in HNSC were estimated using the GEPIA2 (http://gepia2.cancer-pku.cn) and visualized with heatmaps. Enrichment analyses used the LinkInterpreter module, with the 'Overrepresentation Analysis' using positively correlated transcripts ranked with the *p*-value. The ranking criteria for 'Gene Set Enrichment Analysis' was* p*-value, and 500 simulations were performed. Enriched terms were further processed using 'Affinity propagation' to reduce redundancy. Clusters with a *p*-value < 0.05 were considered statistically significant.

### Tumor immune microenvironment analysis

TIMER 2.0 and TISIDB (http://cis.hku.hk/TISIDB/) databases characterized the correlations between *ALDH2* levels and HNSC immune microenvironment, immune signatures, immune cell infiltration, and expression levels of immune-related transcripts. The TIMER platform (https://cistrome.shinyapps.io/timer/) accessed the copy number alteration of *ALDH2* and the 6 representative immune cell infiltrations by HPV status. We further estimated the abundance of 22 CIBERSOFRT immune cells in bulk tumors with different *ALDH2* levels using TIMER 2.0. By HPV status, the correlation analysis evaluated the associations of the abundance of specific immune cells and their respective gene markers with *ALDH2* levels. Furthermore, the TISCH (http://tisch.comp-genomics.org/), a scRNA-seq database, was used to explore the *ALDH2* levels of different cells from bulk tumors across 3 GEO cohorts to refine our understanding of the *ALDH2* levels in regulating immune cells infiltrations.

### Correlation between the ALDH2 level and treatment response of anti-cancer agents

We utilized the DepMap (https://depmap.org/portal/) to obtain the *ALDH2* mRNA levels of HNSC cell lines. Subsequently, we attained the cell median lethal dose (IC_50_) of docetaxel, paclitaxel, cisplatin, 5- fluorouracil (5-FU), methotrexate, and cetuximab of these cell lines from Genomics of Drug Sensitivity in Cancer database (https://www.cancerrxgene.org/). We identified 13 HNSC-derived cancer cell lines, including BIRC22, BICR78, CAL-33, CAL-27, Detroit562, FADU, HSC-2, HSC3, HSC-4, OSC-20, SAS, SAT and SCC-4, which were then subjected to further correlation analysis.

### Lentivirus infection, quantitative RT-PCR and cell viability assay

Lentivirus control (pLKO-1-shLuc967) and shALDH2 viral supernatant (TRCN0000026430) were purchased from the National RNAi Core Facility (Taipei, Taiwan). Viral supernatants were used to infect DOK cells with 8 μg/mL polybrene for 72 h. The stable DOK/shLuc and DOK/shALDH2 cell lines were selected with 2 μg/ml puromycin. The HNSC-derived cell lines, DOK, HSC-3, CAL-27, TW2.6, TW1.5, CA922, and SAS (5 × 10^5^ cells/dish) were plated on the 6-cm culture dishes in a complete medium and incubated overnight. Next, total RNA was extracted for quantitative reverse-transcription PCR (qRT-PCR) using SYBR chemistry. Primers *ALDH2*-forward: 5'-CATGGACGCATCACACAGGG-3' and reverse: 5'-CTTGCCATTGTCCAGGGTCT-3'; and *ACTB*: 5'-AGAAAATCTGGCACCACACC-3' and reverse: 5'-AGAGGCGTACAGGGATAGCA-3'. Cells (2,000 cells/well from DOK, TW2.6, SAS, DOK/shLuc, and DOK/shALDH2) were seeded in 96-well plates. The cell viability was determined by 3-(4,5-dimethylthiazol-2-yl)-2,5-diphenyltetrazolium bromide (MTT) assay. The mean ± standard deviation (SD) of three independent experiments is presented as the results, with differences between the two groups determined by the Student's *t*-test. A significance level of *P* < 0.05 is considered statistically significant and indicated with an asterisk (*).

### Western blot

The BCA protein assay kit (PierceTM BCA Protein Assay Kit; Thermo Fisher, IL, USA) was used to determine the protein concentration after proteins were extracted using RIPA buffer. Proteins were put onto SDE-PAGE gel (10%) from cell lines. After being transferred to a PVDF membrane, blocking was carried out using 5% BSA in PBST. The antibodies were directed against ALDH2 (1:1000, GTX101429, Genetex) and β-actin (1:5000, # A5441, Sigma).

## Results

### The genetic landscape of ALDH2 in HNSC

**Figure 1:**
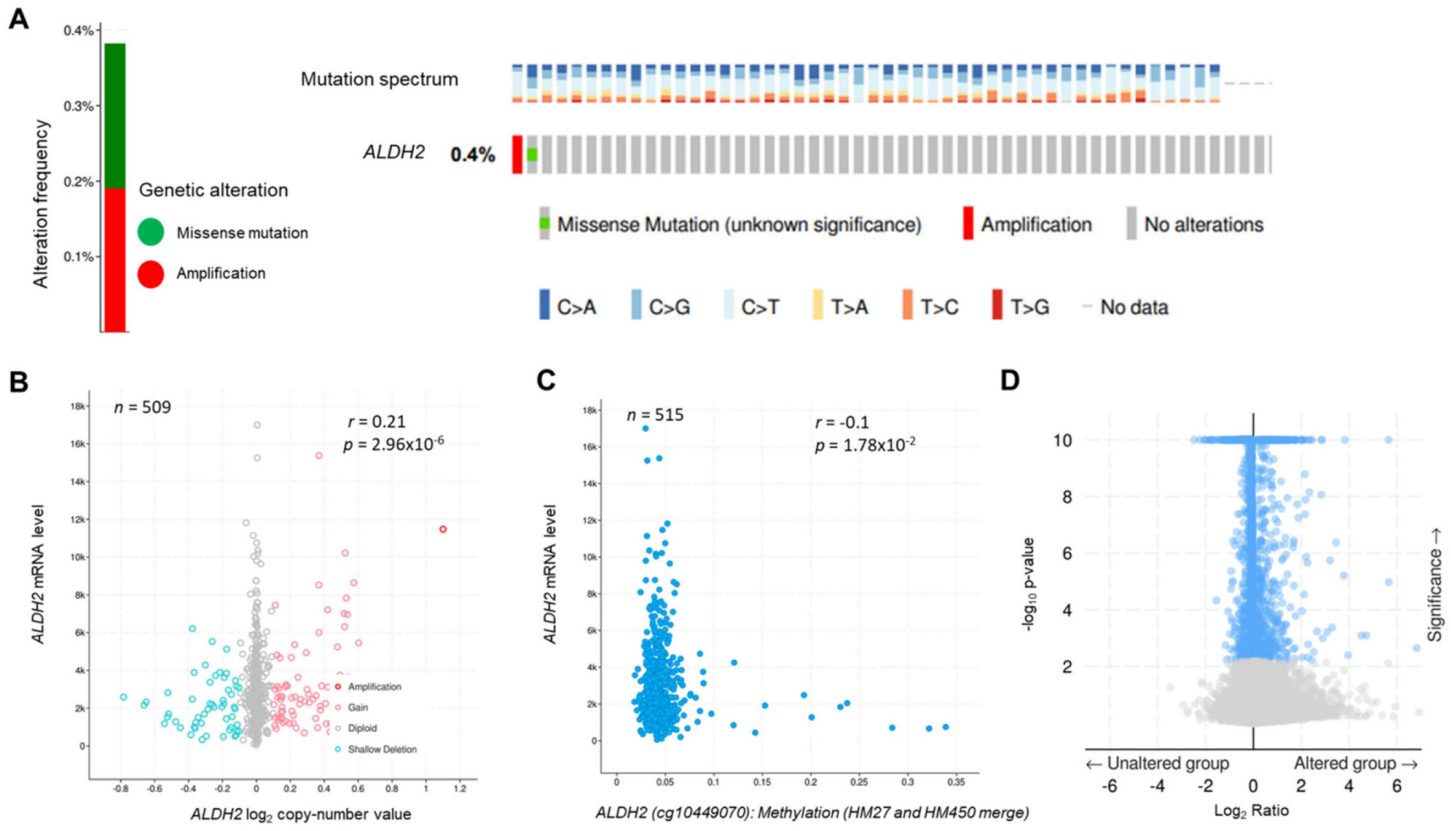
** The genetic alteration and genetic association analysis of *ALDH2* in TCGA-HNSC cohort.** (A) cBioPortal displays various types and frequencies of *ALDH2* genetic alterations in 523 HNSC patients. (B) The correlation between *ALDH2* mRNA level (RNA-Seq by Expectation-Maximization: RSEM) and its log_2_ copy-number value (*n* = 509). (C) The association between *ALDH2* levels and their DNA methylation using the Infinium Human Methylation 27 (HM27) and HumanMethylation450 (HM450) arrays. (D) The significantly altered genes associated with the *ALDH2* mRNA level.

The genetic alteration rate of *ALDH2* in the PanCancer cohort is 2.2%, and the most common form is copy number amplification (**Fig. S1A**). In HNSC (TCGA) cohort, the altered rate is 0.4%, with one missense mutation (location: A245S) and one copy number amplification (**Fig. 1A**). **Fig. 1B** showed there were 74 cases with DNA gain (+1, low-level gain) and 53 cases with DNA shallow deletion (-1, shallow loss); *ALDH2* mRNA level was positively correlated with its Log_2_ copy-number values (*n* = 509, *r* = 0.21, *P* = 2.96×10^-6^, Pearson analysis). **Fig. 1C** revealed the *ALDH2* level negatively associated with the DNA methylation status at the CpG site cg10449070 (*n* = 515, *r* = -0.1, *P* = 1.78×10^-2^). These findings suggested that genetic and mutations epigenetic changes contribute to the decreased *ALDH2* mRNA levels in HNSC tissues. Furthermore, the *ALDH2* alteration significantly correlated to the expression levels of another 1047 transcripts **(Fig. 1D)**. Higher *ALDH2* levels slightly correlate to lower mutation counts (*r* = -0.1, *P* = 2.59×10^-2^, Pearson analysis) (**Fig. S1B**). Further examinations revealed positive associations between tumor mutation burden (TMB) and microsatellite instability (MSI) scores (MSI MANTIS score:* r* = 0.38, *P* = 4.85×10^-19^; MSI SENSOR score: *r* = 0.47, *P* = 2.67×10^-29^) (**Fig. S1C-S1D**). Moreover, low *ALDH2* levels are significantly associated with higher TMB (*r* = -0.12, *P* = 8.17×10^-3^) (**Fig. S1E**).

### Downregulation of the ALDH2 level in HNSC

**Figure 2:**
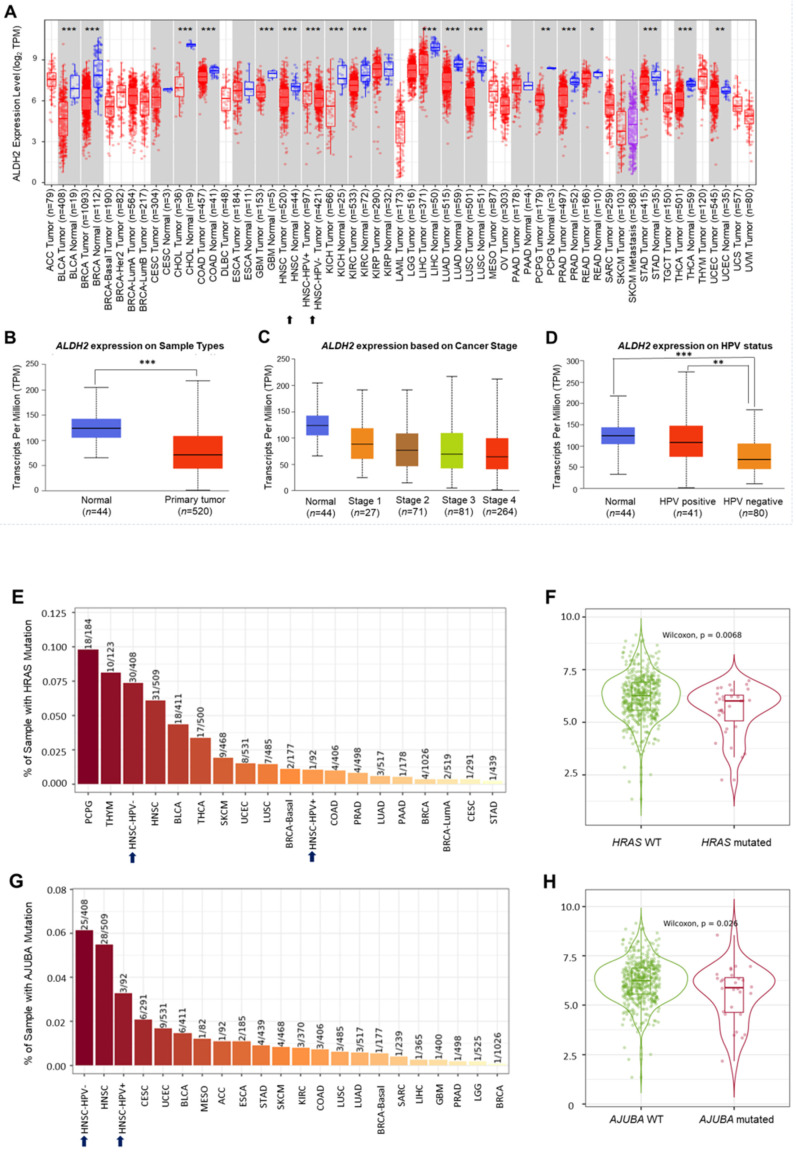
** The *ALDH2* mRNA levels in HNSC patients.** (A) TIMER 2.0 displays the differential expression levels of *ALDH2* in tumor and adjacent non-tumor tissues across all cancer types in TCGA. The asterisk labels statistical significance. (B) The UALCAN platform indicates that the *ALDH2* level in tumors (*n* = 520) is significantly lower than in normal tissues (*n* = 44) in the TCGA-HNSC cohort. (C) The *ALDH2* levels in tumor tissues across different stages. (D) The *ALDH2* levels in HPV+ (*n* = 41), HPV- (*n* = 80) tumors, and normal tissues. (E, G) TIMER 2.0 demonstrates the frequencies of *HRAS* and *AJUBA* mutations in HPV- and HPV+ HNSCs. (F, H) *ALDH2* mRNA levels in *HRAS*- and *AJUBA*-mutated HNSCs compared to their wild types. Statistical significance: *P < 0.05, **P < 0.01, ***P <0.001.

Analysis of the TCGA database showed significantly lower *ALDH2* mRNA levels in the bulk tumors compared to the normal samples in 17 different cancers (**Fig. 2A**). Specifically, in HNSC (*n* = 520), the *ALDH2* levels were significantly downregulated in tumors compared to unpaired healthy tissues (**Fig. 2B**), consistent across the different overall stages (**Fig. 2C**). The *ALDH2* levels were verified in the data from gene chips (**Fig. S2A-S2B**) and RNA-Seq (**Fig. S2C-S2D**) using the TNMplot, where the *ALDH2* levels were higher in normal tissues at each cutoff value. Further, analysis by HPV status (*in situ* hybridization to p16, data from UALCAN) found that the *ALDH2* level of HPV+ HNSC is higher than that of the HPV- HNSC, and there is no significant between HPV+ tumors (*n* = 41) and normal tissues (*n* = 44) on the *ALDH2* mRNA level (*P* = 5.75×10^-1^, **Fig. 2D**). To further explore the potential impacts of the most frequently altered transcripts in the HNSC/TCGA cohort on the *ALDH2* mRNA levels by HPV status, we obtained the top 30 mutated genes from the cBioPortal (**Table S1**). Regarding gene mutations, we found that there are more mutated *HRAS* in the HPV- HNSC (7.35% vs. 1.08%), and HNSC individuals carrying mutated *HRAS* show significantly lower *ALDH2* levels than those without mutations (*P* = 6.8×10^-3^, **Fig. 2E-2F**). We also found a significant difference in the *ALDH2* level between *AJUBA* mutants and wild-type HNSC cases (6.13% vs. 3.26%, **Fig. 2G-2H**). These observations suggested that the difference in *ALDH2* levels between these subpopulations may be attributed to its correlation with the most prevalent altered transcripts in the HNSC/TCGA cohort.

### A high ALDH2 level positively correlates to favorable prognostic variables in HNSC

The mean age of the 490 HNSC patients (360 males, 130 females) was 61.03 years (ranging from 19 to 90), with an average follow-up of 30.34 months. Three-hundred patients (61.2 %) had oral cavity cancers, with the tongue being the most common site (41.6%). Among the other variables, the majority of the patients were HPV- HNSC (83.1%), active/ever drinkers (66.1%) or smokers (62.2%), and stage III/IV (78.4%). The available cases of perineural invasion (PNI) and lymphovascular invasion (LVI) were 344 (70.2%) and 333 (67.9%), respectively. As shown in **Table 1**, low *ALDH2* levels correlated to oral cavity subsite (*P* = 4×10^-4^), HPV- status (*P* = 1×10^-4^), presence of PNI (*P* = 1.77×10^-2^), and advanced AJCC T classification (*P* = 1.5×10^-3^) in HNSC compared to the normal ones. Further categorization of 490 patients into two subgroups (*ALDH2*-low, *n* = 312 vs. *ALDH2*-high, *n* = 182) by taking the mean *ALDH2* as the cutoff found that *ALDH2*-high subgroup remains significant association with HPV+ status (*P* = 3×10^-5^), negative PNI (*P* = 3.31×10^-2^), and early AJCC T stage (*P* = 2×10^-4^) yet marginally inverse association with the oral cavity anatomic subsite (*P* = 8.34×10^-2^) (**Table 1**). The T stage and HPV status remain the influential factors related to the *ALDH2* level when using medium *ALDH2* values as the cutoff value (**Table 1**). The univariate analysis demonstrated that early T stages (T1/T2) were significantly correlated to high *ALDH2* levels in both oral cavity cancer (*n* = 300,* P* = 1.7×10^-3^) and non-oral cavity cancer (*n* = 190,* P* = 4.89×10^-2^, student *t*-test) groups (**Table 2**). Additional subgroup analysis by HPV status revealed that the association continued in the HPV- group (*n* = 407) regardless of the form of study: mean ± SD (11.18 ± 6.94 of T1/T2 vs. 9.62 ± 7.15 of T3/T4; *P* = 3.41×10^-2^, student *t*-test) or dichotomized groups by the medium value (65/218, 29.8% vs. 79/189, 41.8% of T1/T2 patients; *P* = 1.16×10^-2^, chi-square test) (**Table 3**). However, no significant correlation was found between the *ALDH2* level and T stage in the HPV+ group (*n* = 69, *P* = 3.375×10^-1^, Mann-Whitney U test) (**Table 3**).

### The ALDH2 protein level of HNSC from tissue microarrays and human specimens

**Figure 3:**
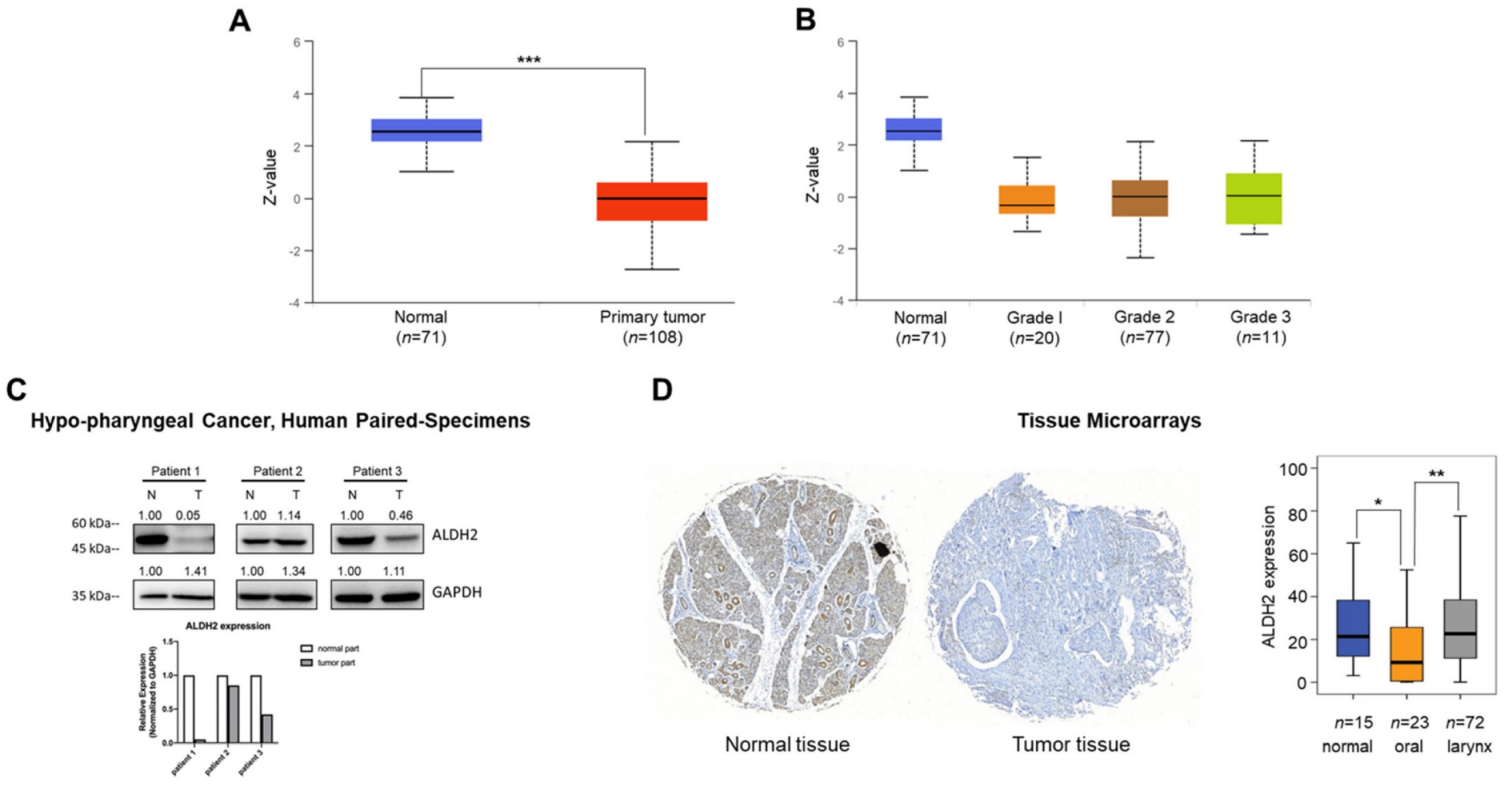
** Different sources show the ALDH2 protein levels in HNSC patients.** (A) The UALCAN platform demonstrates the ALDH2 protein level in tumors (*n* = 108) and non-tumor tissues (*n* = 71). (B) The ALDH2 protein levels in different histology grades of tumors. (C) Western blot analysis showed the ALDH2 protein levels in tumor and adjacent nontumor tissues in three patients with hypopharyngeal cancer. (D) Immunohistochemistry on the tissue microarrays displayed the ALDH2 protein levels in normal tissues (*n* = 15), oral cancer (*n* = 23), and laryngeal cancer (*n* = 72), and representative images scored by Histoquest software.

We next probed the ALDH2 protein level of the tumor and normal tissues of HNSC patients. The CPTAC prediction through the UALCAN platform showed markedly lower ALDH2 protein levels in tumorous (*n* = 108) than those in normal tissues (*n* = 71, *P* = 2.04×10^-41^) (**Fig. 3A**), and the trend continued regardless of the tumor grade (**Fig. 3B**). Western blot analysis revealed that the ALDH2 level of hypopharyngeal cancer tissues tends to be lower than those in paired non-cancer tissues (*n* = 3, **Fig. 3C**), suggesting the trend is consistent with those from the omic prediction from the HNSC/TCGA cohort. We further analyzed whether ALDH2 protein levels impact anatomic subsites in two HNSC tissue microarrays (HN721, HN802c). The mean age of the TMAs was 51.91 years (ranging from 15 to 78). After excluding 22 pathological non-SCC, 12 without available tissues, and 2 without clinical data for evaluation, we found no significant difference in the average ALDH2 protein levels between 101 tumor specimens and 15 normal samples (24.46 ± 21.73 vs. 28.70 ± 21.60, *P* = 0.3921, Mann-Whitney U test). However, we found that the ALDH2 levels were downregulated in the oral cavity cancers (*n* = 23, 14.28 ± 16.51) compared to healthy tissues (*P* = 2.15×10^-2^), whereas no significant difference compared to laryngeal cancer (*n* = 72, 27.11 ± 21.61; *P* = 7.49×10^-1^) (**Fig. 3D**). **Table S2** demonstrated that the ALDH2 protein level was also inversely correlated to histology grade (*P* = 3.7×10^-3^). Therefore, these observations support that ALDH2 potentially plays roles in oncogenesis, cancer progression, cellular differentiation, and its impacts related to the anatomic subsite.

### High ALDH2 protein levels associated with better survival rates in HNSC patients

**Figure 4:**
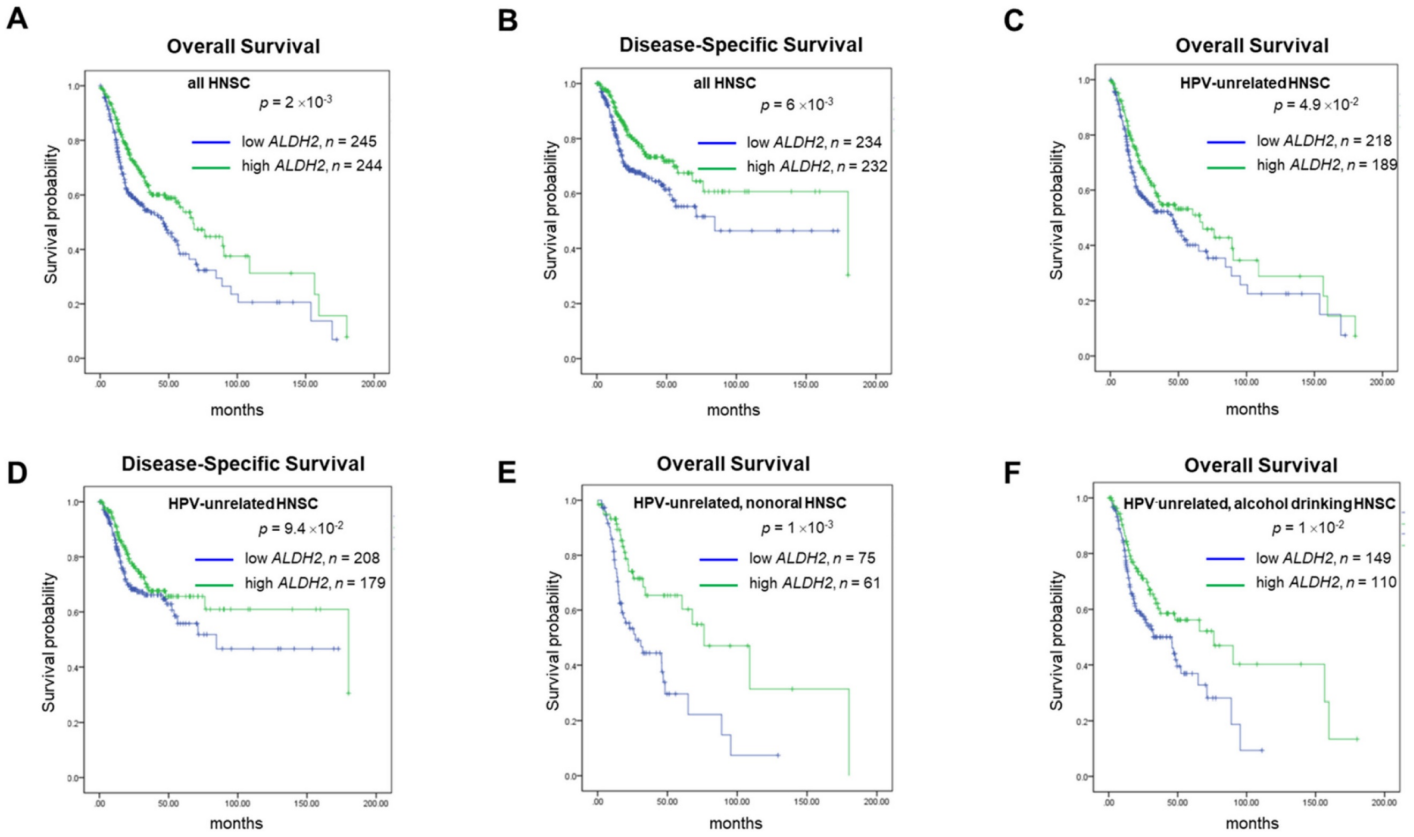
** The survival analysis of *ALDH2* mRNA levels in HNSC patients based on HPV condition, anatomic subsite, and alcohol consumption status.** Kaplan-Meier estimator shows the (A, B) 15-year overall survival and disease-specific survival rates in patients with high and low *ALDH2* levels. (C, D) 15-year Overall survival (OS) and disease-specific survival (DSS) of HPV- patients with medium was used as the cutoffs. (E) HPV-/non-oral HNSC patients. (F) HPV-/alcohol drinking patients. The medium *ALDH2* level of 9.043 was used as the cutoff.

For prognosis, patients with higher *ALDH2* levels (*ALDH2*-H subgroup) conferred better 15-year overall and disease-specific (DSS) rates compared to those with lower *ALDH2* levels (*ALDH2*-L subgroup), with a medium *ALDH2* level of 9.043 as the cut-off value (**Fig. 4A-4B**). Survival analyses by HPV status (**Fig. 4C-4D**) showed the difference in overall survival (OS) persisted in HPV-unrelated HNSC (*P* = 4.9×10^-2^). Further analyses by the anatomic subsite demonstrated that the effect of *ALDH2* level on OS was statistically significant in the non-oral anatomic subsite (*P* = 1×10^-3^) (**Fig. 4E**). The results also suggested that alcohol consumption status may affect the impact of the *ALDH2* levels on OS in HPV-HNSC patients with active/ever alcohol drinking. Specifically, the *ALDH2*-L subgroup had worse 15-year OS than the *ALDH2*-H subgroup in these patients (*P* = 1×10^-2^) (**Fig. 4F**).

### ALDH2 significantly associated with ALDH6A1 in the interacted gene/protein networks

**Figure 5:**
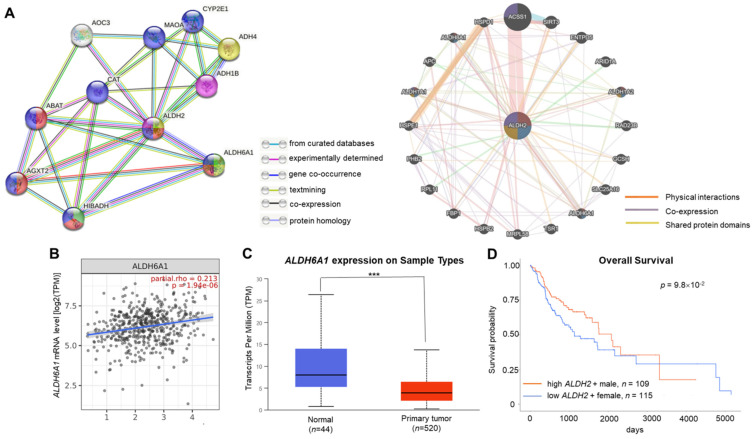
** The gene and protein interacting networks with ALDH2.** (A) STRING (left panel) shows the interaction between ALDH2 and ALDH6A1, which was experimentally determined. GeneMANIA (right panel) analysis using the networks including 'physical interactions', 'co-expression', and 'shared protein domains' highlight the genes, including ALDH6A1, with functions enriched in 'oxidorectase activity', 'small molecule binding', and 'catalytic activity'. (B) The correlation between *ALDH2* and *ALDH6A1* mRNA levels (TIMER 2.0) (C) The *ALDH6A1* levels in HNSC tumor and normal tissues (UALCAN). (D) The survival curves of female patients with low ALDH6A1 and male patients with high expression of ALDH6A1 (UALCAN).

The GeneMANIA and STRING databases found that ALDH2 significantly correlated to ALDH6A1 at both the transcription and translational levels. The STRING analysis revealed that ALDH6A1 robustly interacts with ALDH2 with a combined score of 0.961, while GeneMANIA showed their physical interactions, co-expression, and shared protein domains (**Fig. 5A**). The relationship between *ALDH2* and *ALDH6A1* was validated by TIMER 2.0 with a positive correlation between these transcripts (partial *rho* = 0.213, *P* = 1.94×10^-6^) (**Fig. 5B**). The downregulation of *ALDH6A1* in tumor tissues (*P* = 1.61×10^-5^) (**Fig. 5C**) and the marginally better overall survival of male HNSC patients with high *ALDH6A1* level than females with low *ALDH6A1* level (*P* = 9.8×10^-2^, **Fig. 5D**) suggest that ALDH2 and ALDH6A1 may contribute to differences in ALDH2 expression between normal and tumor tissues and survival in HNSC.

### The functional network of the ALDH2 in HNSC

**Figure 6:**
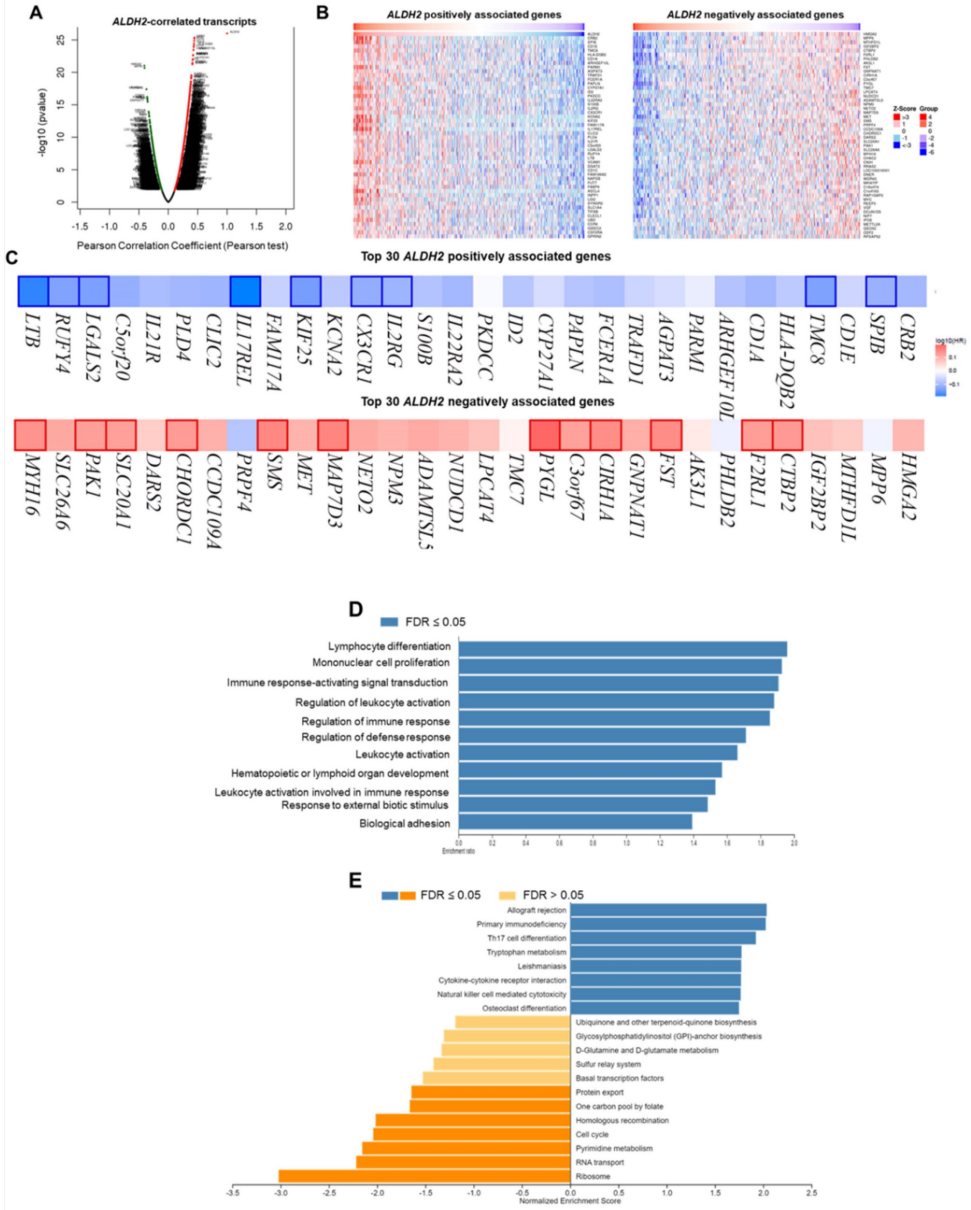
** The LinkedOmics database shows the co-expressed transcripts with *ALDH2* and enrichment analysis in HNSC.** (A) A volcano plot displays the *ALDH2*-coexpressed transcripts. Among them, 5009 reached statistical significance (red: positive correlation; green: negative correlation). (B) The heatmaps indicate the top 50 positively and negatively correlated transcripts. (C) The top 30 positively and negatively correlated transcripts in the overall survival of HNSC patients (blue color, negative prognostic predictor; red color, positive prognostic predictor). The bordered genes are statistically significant differences in survival. (D) The bar diagram shows the co-expressed transcripts with significant enrichment in GO annotations for Biological Process, while (E) GSEA identifies enriched KEGG pathways.

**Figure 7:**
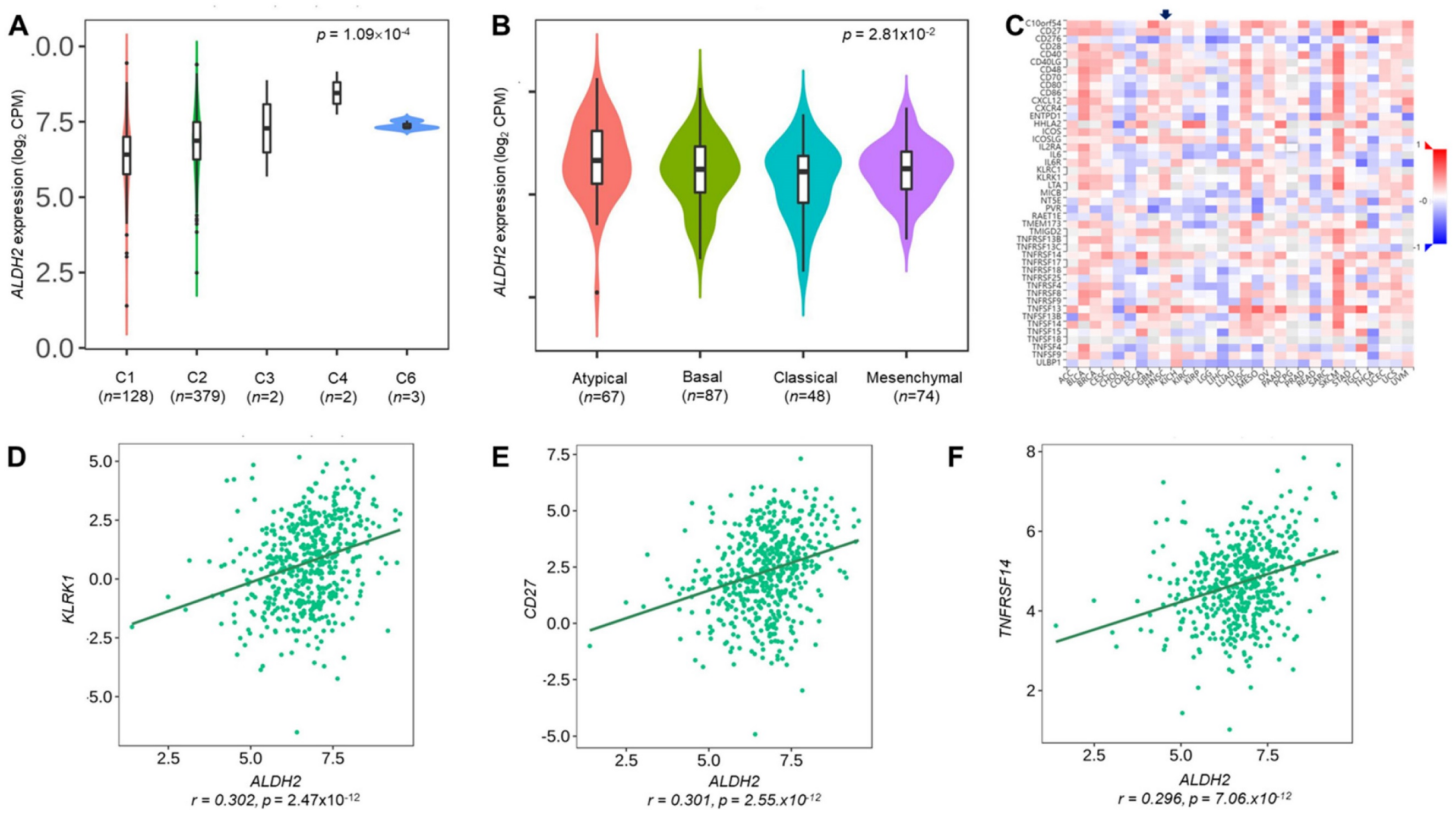
** The relationships between the *ALDH2* level and immune subtype, molecular subtype, and immunostimulatory molecules.** The TISIDB database displayed the levels of *ALDH2* in the TCGA-HNSC cohort (*n* = 522) in different (A) immune subtypes and (B) molecular subtypes. (C) A heatmap shows the association between the *ALDH2* levels and representative immunostimulators in various cancer types, where an arrowhead indicates the significance of HNSC. (D-F) The top three correlated transcripts were *KLRK1*, *CD27*, and *TNFRSF14*.

We employ the LinkedOmics to explore the co-expression network of *ALDH2* in the HNSC/TCGA cohort (*n* = 517). **Fig. 6A** shows that 5009 transcripts (3431 positively and 1578 negatively correlated) were altered with *ALDH2* fluctuation (*P* < 0.01, FDR < 0.01). Among the top 30 altered transcripts (**Fig. 6B**), 9 positively and 12 negatively correlated transcripts were significantly predictive of the overall survival of HNSC (**Fig. 6C**), indicating a strong impact of the *ALDH2* network on the pathogenesis of HNSC. Since the above 21 transcripts show strong correlations to the *ALDH2* levels, implying their potential roles to contribute to the survival difference through the co-regulation with the *ALDH2* level. Gene ontology (GO) enrichment analysis on the co-expressed transcripts (**Fig. 6D**) demonstrated that most enriched terms at the biological process were related to the immune-related clusters. GSEA showed enriched KEGG pathways (**Fig. 6E**), including 'Th17 cell differentiation', 'natural killer cell-mediated cytotoxicity', and 'cytokine-cytokine receptor interaction'. These observations suggest that *ALDH2* may be an immune-related factor in HNSC. Thus, we applied the TISIDB dataset to probe the association of the *ALDH2* level with different immune signatures. As shown in **Fig. 7A**, the C2 subtype (IFN-gamma dominant, *n* = 379) exhibits a higher *ALDH2* level than the C1 subtype (wound healing, *n* = 128) (*P* = 1.09×10^-4^, Kruskai Wallis test). Regarding the molecular subtypes, the *ALDH2* level in the atypical subtype was higher than those of basal -, classical-, and mesenchymal -subtype samples (**Fig. 7B**, *P* = 2.81×10^-2^). We also found *ALDH2* positively correlates to the levels of 15 immuno-stimulators with Spearman's correlation coefficient greater than 2 (**Fig. 7C**). The top 3 transcripts were *killer cell lectin like receptor K1* (*KLRK1*) (*r* = 0.302, *P* = 2.47×10^-12^), *TNF Receptor Superfamily Member 7* (*CD27*) (*r* = 0.301, *P* = 2.55×10^-12^), and *TNF Receptor Superfamily Member 14* ***(****TNFRSF14*) (*r* = 0.296, *P* = 7.01×10^-12^). These findings suggested the co-regulation of different immune-related molecules and *ALDH2* (**Fig. 7D-7F**).

### The relationship between the ALDH2 level and tumor immune infiltration

**Figure 8:**
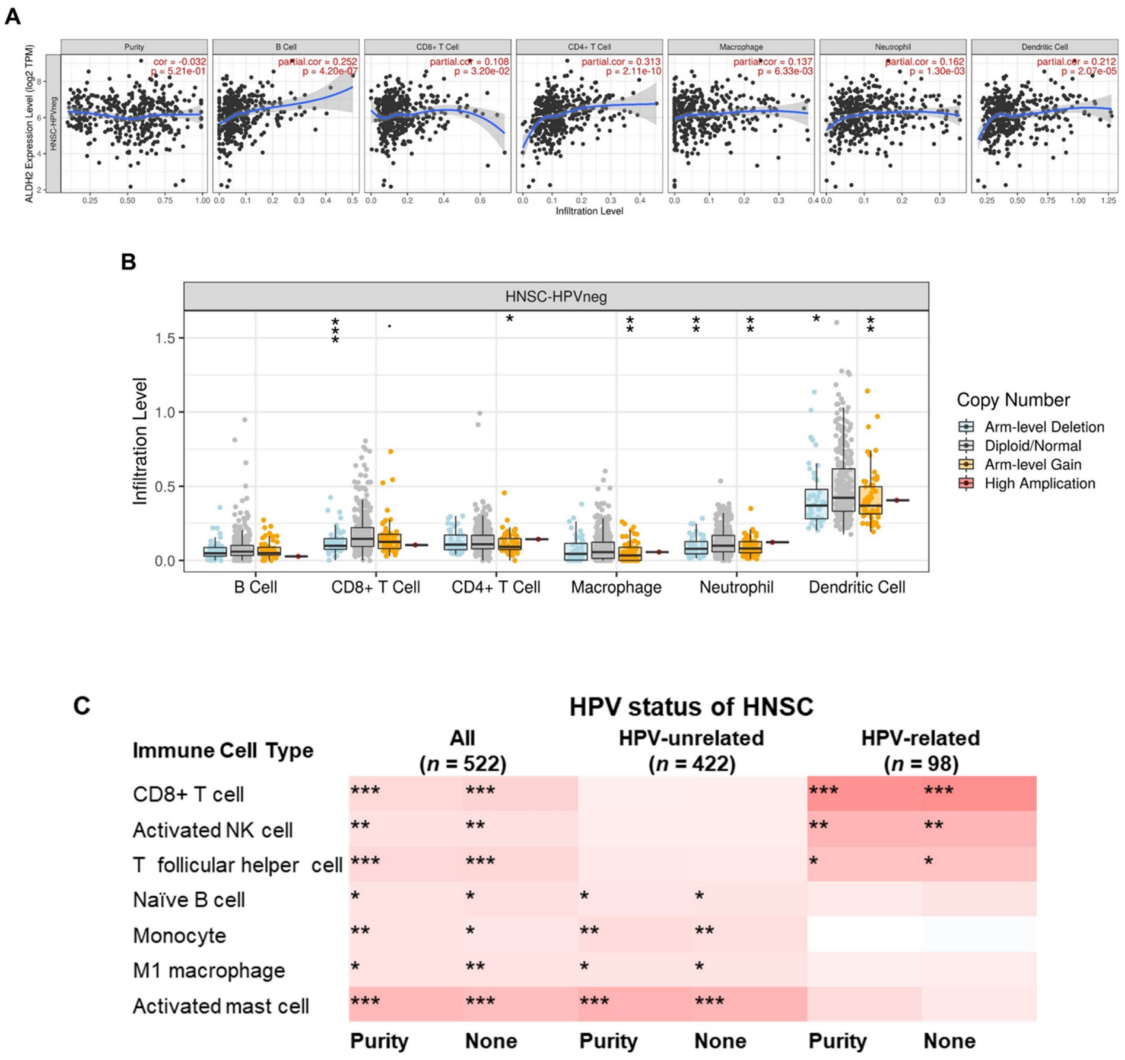
** The correlations between the *ALDH2* levels and infiltration levels of tumor-infiltrating immune cells in HNSC patients by HPV status.** (A) The TIMER database revealed the associations between the tumor-infiltrating levels of six immune cell types and tumor *ALDH2* levels following adjustment for tumor purity. (B) The relationship between immune cell infiltrates and *ALDH2* copy number alteration. (C) The TIMER 2.0 data portal demonstrated the link between the infiltration levels of 7 CIBERSORT immune cells and tumor *ALDH2* levels across overall- (*n* = 522), HPV- (*n* = 422), and HPV+ (*n* = 98) HNSCs. Statistical significance: *P < 0.05, **P < 0.01, ***P < 0.001.

To further explore the immunologic differences by *ALDH2* variations in the HNSC/TCGA cohort, we first investigated the impact of the *ALDH*2 level on six immune cells from the TIMER database. The results showed a positive correlation between the *ALDH2* level and immune cell infiltrates (**Fig. 8A**), and *ALDH2* copy number alterations (CNA) impacted the infiltration of CD8+ T cells, macrophages, neutrophils, and dendritic cells (**Fig. 8B**). *ALDH2* CNA additionally affected the infiltration of CD4+ T cells, and *ALDH2* arm-level deletion was associated with lower infiltrates of CD8+ T cells, neutrophils, and dendritic cells in the HPV-HNSC (**Fig. S3**). To further examine the effects of HPV status on 22 immune infiltrations by the *ALDH2* level, we intersected the results from the TISIDB and TIMER 2.0 (CIBERSORT algorithm) to identify the meaningful infiltrated immune cells. We found positive correlations between the *ALDH2* level and the abundance of CD8+ T, naïve B, monocytes, M1 macrophages, activated NK cells, T follicular helper (Tfh), and activated mast cells in tumors, regardless of the HPV status (**Fig. 8C**). There was also a relationship between *ALDH2* and the proportions of naïve B cells, monocytes, M1 Macrophages, and mast cells in HPV-HNSC, with the infiltrations of CD8+ T, activated NK, and Tfh cells in HPV+ HNSC (**Fig. 8C**). The correlations between respective gene markers of immune cells and the *ALDH2* levels further strengthened the above relationships (**Fig. S4A**). The TISCH database indicates that the *ALDH2* levels in macrophages/monocytes are higher than in the cancer cells in all 3 cohorts, suggesting that the *ALDH2* levels in the immune cells play a predominant role (**Fig. S4B**). However, only in GSE139324 (GEO, NCBI), B cells express *ALDH2*, implying that other mechanisms beyond *ALDH2* expression in immune cells in regulating immune cell infiltration. These findings suggested that the *ALDH2* levels may impact aggressiveness in HNSC partly through promoting immune cell infiltration in the tumor microenvironment.

### Low ALDH2 levels associated with reduced chemotherapy responses in HNSC cells

**Figure 9:**
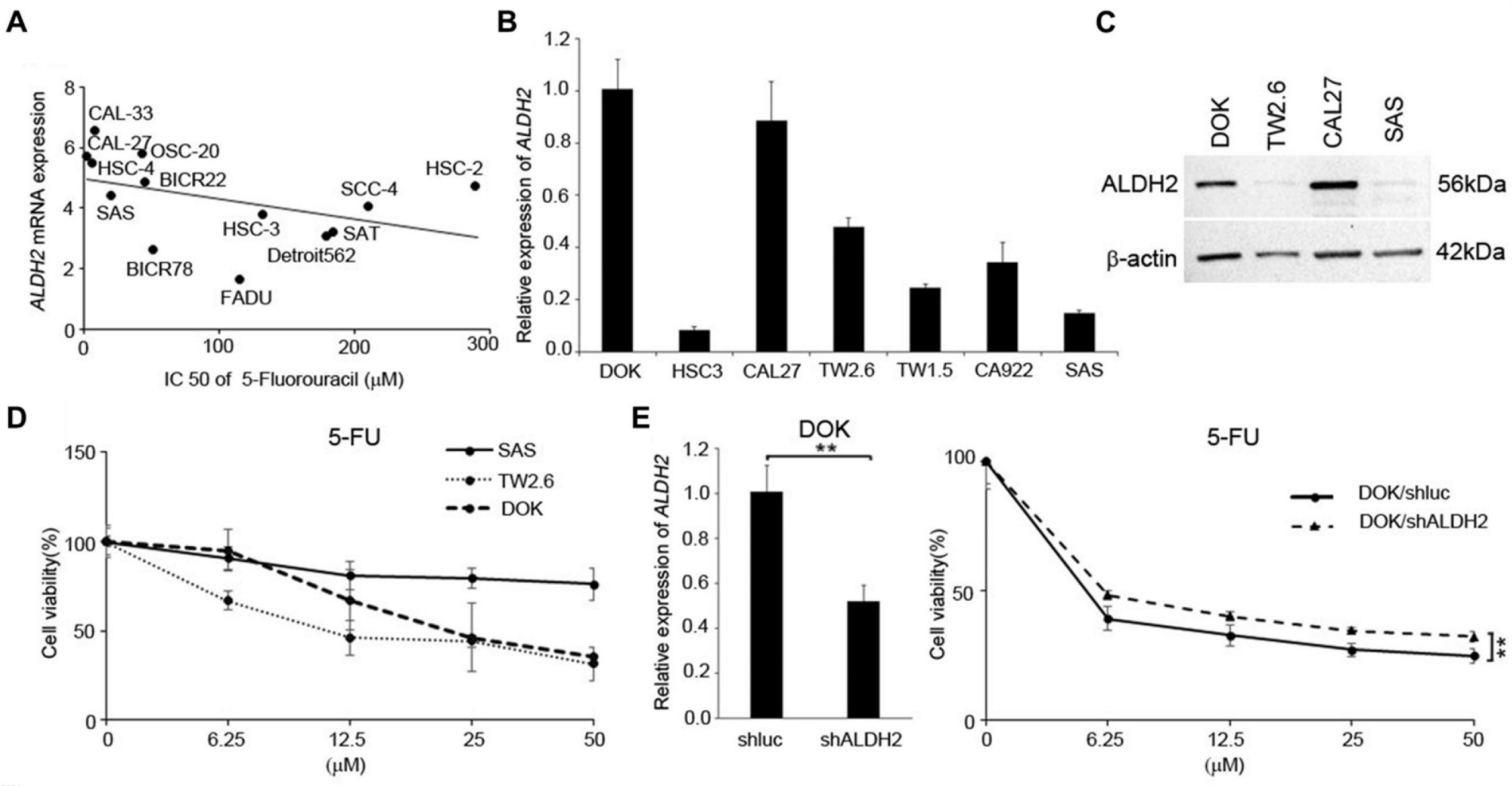
** The *ALDH2* levels affected 5-fluorouracil (5-FU) response in HNSC-derived cells.** (A) Cancer Cell Line Encyclopedia (CCLE) and Genomics of Drug Sensitivity in Cancer (GDSC) databases demonstrate the *ALDH2* levels and the IC_50_ values for 5-FU in 13 head and neck cancer cell lines. (B) Quantitative RT-PCR measured the endogenous *ALDH2* levels in 7 HNSC-derived cell lines. (C) Western blot analysis of HNSC cell lines of DOK-, CAL27- cells (high endogenous *ALDH2* level), TW2.6 cell (medium endogenous *ALDH2* level), and SAS cell (low endogenous *ALDH2* level). (D) MTT assay showed the cell viability of SAS, TW2.6, and DOK cells after treatment with different concentrations of 5-FU. (E) Quantitative RT-PCR (left panel) determined the *ALDH2* levels in DOK/shLuc (control) and DOK/shALDH2 cells. The response of DOK cells to 5-FU treatment (right panel) was evaluated across a final concentration of 50 μM for 72 hours.

We next used the data from several *in silico* studies to examine whether the *ALDH2* levels conferred drug resistance. The Cancer Cell Line Encyclopedia (CCLE) and the Genomics of Drug Sensitivity in Cancer (GDSC) provide the *ALDH2* mRNA levels in 13 HNSC cell lines (**Fig. 9A**) and the IC_50_ values for systemic agents (chemotherapy and molecular-targeted treatment). The systemic agents include docetaxel, paclitaxel, cisplatin, 5-FU, methotrexate, and cetuximab. We observed that the *ALDH2* mRNA levels were negatively associated with the IC_50_ values of 5-FU (*r* = -0.593, *P* = 0.033; **Table 4**). Also, a negative correlation was found between the IC_50_ value of docetaxel, and the *ALDH2* mRNA level, while the prediction model was not statistically significant (*P* = 0.241; **Table 4**). We then enlisted 7 HNSC cell lines with different levels of endogenous mRNA* ALDH2* levels and used SAS (low *ALDH2*), TW2.6 (medium *ALDH*2), and DOK (high* ALDH*2) for further evaluation (**Fig. 9B**). We also examined the expression levels of ALDH2 protein in HNSC cells and observed that the protein expression patterns were consistent with the mRNA levels in HNC cells, as shown in **Fig. 9C**. The MTT assay showed cell viability decreased in DOK and TW2.6 compared with SAS after treatment with 5-FU (**Fig. 9D**). Given the consistent cell viability differences between cell lines across all the concentrations after 5-FU treatment, we knocked down *ALDH2* in DOK cells to evaluate the drug responses. The results showed consistent differences in cell viability between DOK/shLuc and DOK/shALDH2 cells (*P* < 0.01, **Fig. 9E**), suggesting that a high *ALDH2* level bestows better treatment response to 5-FU.

## Discussion

In this study, we applied multi-omics to study the roles of ALDH2 on HNSC. Drug prediction and our *in vitro* studies demonstrated low *ALDH2* levels decreased 5-FU response. The combination of 5-FU and cisplatin as the induction chemotherapy regimen has been a promising strategy for selecting responders to preserve organs in patients with locally advanced laryngeal and hypopharyngeal cancers 15 with a comparable 10-year overall rate (13.8%) compared to that of the surgery-based subgroup (13.1%) 16, suggesting our results providing potential clinical applications. Induction 5-FU and cisplatin was also influential in down-staging oral cancers that may be borderline resectable 17 or unresectable 18,19, though the survival benefit in the unresectable subgroup is controversial 18,19. The metronomic use of Uracil (inhibits the enzymes that metabolize 5-FU)-Tegafur (precursor of 5-FU) was proven to reduce metastasis in advanced-stage oral cancer patients 20. These results suggested the *ALDH2* level might predict chemoselection and the risk of metastasis after 5-FU treatment. The potential mechanisms include immunogenic cell death 21, as higher *ALDH2* levels are correlated with increased infiltration of antigen-presenting cells, and *ALDH2*-associated co-expressed genes are involved in many immune-related processes. However, the association is somehow contradictory to other studies that show increased ALDH2 enzymatic activity can enhance chemoresistance in specific contexts, such as with doxorubicin in renal cells 13 and microtubule inhibitors in lung cancers 14, suggesting the importance of considering the particular condition when studying the role of ALDH2 in drug resistance.

The study showed lower *ALDH2* levels in HNSC tissues were partly related to copy-number alteration and hyper-methylations. Moreover, the *ALDH2* mRNA and protein levels in tumors were influenced by HPV status, potentially through differences in *ALDH2* levels caused by common mutations of the HNSC/TCGA cohort, and differences in an anatomic subsite. We also found that low levels of *ALDH2* are negatively correlated with AJCC T classification and predict worse overall survival in HPV-unrelated HNSC using the medium *ALDH2* level as the cut-off value. Further exploration revealed the negative prognostic impacts of down-regulated *ALDH2* may be related to its association with *ALDH6A1*, lower infiltrations of immune surveillance cells, and the majority of *ALDH2*-associated co-expressed genes that have prognostic significance. Additionally, high* ALDH2* mRNA level was associated with better response to fluorouracil in both in silico and *in vitro* studies.

The *ALDH2* SNP rs671 variant commonly found in East Asians may lead to increased risk for initiation, faster progression, and a worse prognosis of alcohol-related cancers 3,4. Preceding preclinical studies have demonstrated that the ALDH2*2 allele, which encodes a dominant-negative enzyme variant with reduced activity, can alter cancer cells' biological behaviors and phenotypes in various ways 3,4. However, not only are the *ALDH2* variants but *ALDH2* expression has been linked to cancer pathogenesis and progression. The mechanisms behind the repressed *ALDH2* levels include genetic instability 5-7, enhanced cancer stemness 8, and dysregulated immunity 9-11. For genomic instability, a study has shown that *ALDH2* suppression is associated with a higher DNA base excision repair protein (*XRCC1*, *X-Ray Repair Cross Complementing 1*) and worse survival in lung and liver cancers 7. Additionally, ALDH2 deficiency may cause DNA damage through DNA adducts, DNA interstrand crosslinks, DNA double-strand breaks, tandem mutations resulting from the accumulation of acetaldehyde 22, and requiring various DNA damage repair pathways to prevent mutagenesis 22. For the Fanconi anemia (FA) pathway, acetaldehyde has been found to induce monoubiquitination of the FANCD2 subunit of the FANCD2-FANCI complexes to remove DNA interstrand crosslinks 23. *In vivo*, the impaired FA pathway in double-knockout mice (*ALDH2*-/-, *FAND2*-/-) may cause potential cancer initiation even without ethanol administration 5, as other DNA repair processes cannot substitute the FA pathway 6. ALDH2 is also implicated in cancer stemness regulation, as upregulation of ALDH2 has been found to inhibit stemness and migration of lung cancer *in vivo* 8. Many ways have been shown to control ALDH2 levels. Epigenetic transcriptional control represents one of the primary mechanisms. Evidence shows that SET protein, a histone acetylation modulator, interacts with the promoter region of the *ALDH2* gene via the SET NAP domain to downregulate *ALDH2* level in HNSC cell lines 24.

For the difference in *ALDH2* levels between HPV status, we rationale it by the significant difference in *ALDH2* levels between the top-ranked mutation genes and their wild types. Further evidence for this difference was found in the significantly higher levels of ALDH2 transcripts in the atypical subtype that predominantly segregates HPV-related HNSC 2. Collaborate with the findings that low-*ALDH2* levels were significantly associated with the advanced T category in the HPV-negative subgroup, suggesting that *ALDH2* may play a role in HPV-unrelated HNSC. The prognostic significance of *ALDH2* level was further verified that the low-*ALDH*2 subgroup had less favorable overall survival compared to high-*ALDH2* in the HPV-unrelated tumors. The trend persisted in subgroup analyses by anatomic tumor subsite and alcohol consumption status, despite the complexity of treatment strategies and treatment intent by *ALDH2* levels. However, the effects of comorbidities might partially explain the insignificance in overall survival for *ALDH2* levels in HPV-negative HNSC with the anatomic subsite being the oral cavity or without active/ever alcohol drinking. *ALDH2* levels have been implicated in stress-related disorders 25,26, which can affect the comorbidity profile of cancer patients. Confounding may occur in our interpretation of the relationship between ALDH2 levels and overall survival if a patient dies from comorbidity before cancer.

The potential mechanisms of the association of low *ALDH2* with cancer aggressiveness include the possible interaction with another member of the ALDH family, ALDH6A1, based on the gene and protein co-expression network analysis. Downregulation of *ALDH6A1* implicates the initiation and progression of different types of cancer. An *in vitro* and *in vivo* study has shown that the knockdown of *ALDH6A1* can promote cancer growth and reduce response to chemotherapy cisplatin through the negative regulation of the *hepatocyte nuclear factor 4 alpha* (*HNF4α*) in bladder cancer 27. Another cellular study demonstrated that overexpressed *ALDH6A1* transcripts could inhibit the proliferation and migration of colon cancer through the inhibition of the RAS/RAF/MEK/ERK pathway with the inhibitor MCP110 28. Our results may rationale the assumption that *ALDH6A1* and *ALDH2* may work together to promote better survival outcomes in HNSC because both were reduced in HNSC tissues, and decreased *ALDH6A1* was marginally associated with poorer survival in female patients. However, further research is needed to understand the machinery for this association.

An additional reason is *ALDH*2 level may regulate immunity surveillance, and the effect varies depending on the type of cancer 10,11. In liver cancer, *ALDH2* blocks *nuclear factor erythroid 2-related factor 2* (*Nrf2*) activation by suppressing reactive oxygen species to increase autophagy and repress immune escape 10. However, in colon cancer, ALDH2 stabilizes the alcohol-induced ligand programmed cell death receptor 1 (PD-L1) through inhibiting E3 ubiquitin-mediated proteasome degradation, resulting in an increase in T cell infiltration in cancer cells with low ALDH2 11. In HNSC, we observe a positive association between *ALDH2* expression and the infiltration of monocytes, M1 macrophages, and naïve B cells in HPV-unrelated cancers. From the perspective of *ALDH2* on the macrophage, we additionally found a high abundance of *ALDH2* transcripts in macrophages across 3 different cohorts and significant correlations between M1 macrophage markers and *ALDH2* levels in HPV-unrelated HNSC. Since *ALDH2* simultaneously correlates with monocyte and M1 macrophage infiltrates, we speculate that *ALDH2* might be involved in macrophage polarization. The finding supports our hypothesis that *ALDH2* also positively correlates with molecules involved in promoting macrophage M1 polarization, such as *myeloid differentiation primary response 88* (*MyD88*), *Tumor necrosis factor receptor 1* (*TNFRSF1A*), and *Phosphatase and tensin homolog* (*PTEN*) in HPV-unrelated HNSC, but not in HPV-related subgroup (**Fig. S5A and B**). The MyD88, for instance, is an essential adaptor protein for Toll-like receptor (TLR) signaling, which can suppress M2 gene expression in tumor-associated macrophage (TAMs) and promote tumoricidal M1 phenotype through the activation of TLR4/MyD88/NF-κB pathway 29. Type 1 TNFR signaling, on the other hand, is a crucial negative regulator of M2 TAMs. In mice lacking *TNFR*, there was a substantial reduction in most M1 gene expression, with a concomitant increase in tumor size 30. Additionally, *PTEN* has a significant role in the differentiation of M1 macrophages, as shown in research on mice with a myeloid-specific *PTEN* knockout. They exhibited Akt activation downstream of *PTEN* deficiency appears to contribute to the bias towards M2 activation in these macrophages, resulting in a reduction of pro-inflammatory TNF-α and an increase of anti-inflammatory IL-10 levels upon exposure to TLR ligands 31. These findings suggested M1 macrophage abundance in the bulk tumor may be mediated through its *ALDH2* levels to suppress cancer progression through TAM-mediated mechanisms 32.

Our results also demonstrated a positive correlation between *ALDH2* levels and the presence of tumor-infiltrating B cells (TIL-Bs), essential adaptive immune cells often found in cancers caused by carcinogens and viral infections 33. TIL-Bs have various antitumor immune mechanisms, including producing tumor-specific antibodies and inducing antibody-dependent cellular cytotoxicity, inducing tumor apoptosis through granzyme B production, and acting as antigen-presenting cells 34. Previous research has shown that TIL-B aggregates and their gene signatures are associated with improved outcomes in cancer patients despite the heterogeneity of TIL-Bs in the tumor microenvironment (TME) 35. Additionally, intratumoral tertiary lymphoid structures (TLSs), where B cells differentiate, have been linked to better prognosis and response to immunotherapy in numerous investigations 33,36. In the case of HNSC, a study using single-cell RNA sequencing demonstrated both TLSs with germinal centers (GC) and TIL-Bs with transcriptional signatures of GC are increased in patients with HPV-related HNSC and are associated with improved outcomes 37. They further demonstrated SEMA4A, a membranous glycoprotein that facilitates the immune aggregates via TIL-Bs interaction with endothelial and T cells 38, is associated with the shift from naïve to GC B cells 37. *SEMA4A* level was increased in GC TIL-Bs compared to other TIL-B subtypes and characterized their levels in HPV-positive cases distinctly from those in HPV-negative cases 37. These findings suggest that targeting effective molecules inducing TLS formation and regulating TIL-Bs in TME could potentially enhance the humoral arm of the antitumor immune response in HNSC. Our results found positive correlations between *ALDH2* and B cell infiltrates in patients with HPV-HNSC that can partially explain the negative relationship of *ALDH2* level with HNSC aggressiveness. However, further investigation is needed to understand the mechanisms contributing to the relationship between *ALDH2* expressions and B cell infiltration within the tumor immune microenvironment.

## Conclusion

Taken together, our results found that low tumor *ALDH2* levels were linked with copy-number alteration and hyper-methylation, as well as being negatively associated with T stage and predicting poor overall survival in the HPV-unrelated HNSC. We also observed differences in *ALDH2* levels between tumor and normal tissues based on HPV status, anatomic subsite, and potential interactions between ALDH2 and ALDH6A1. Our analysis on co-expressed genes suggested that *ALDH2* may play a role in the tumor immune microenvironment, impacting the response to antitumor agents. Specifically, higher *ALDH2* levels correlate with higher infiltration rates of different immune cells by HPV status and better response to 5-FU. These results suggest that ALDH2 could be a potential biomarker in HNSC, particularly in the HPV-unrelated subgroup, although further studies are required to confirm these findings.

## Supplementary Material

Supplementary figures and tables.

## Figures and Tables

**Table 1:** The association of *ALDH2* level with the clinical variables

Characteristics			Mean ± SD			Mean value				Medium value		
		*n* = 490	*ALDH2* level	*p* - value		*ALDH2*-L	*ALDH2*-H	*p* - value		*ALDH*2-L	*ALDH2*-H	*p* - value
Age				0.4464				0.8771				0.3841
	< 60	216	11.72 ± 10.46			137	79			113	103	
	≥ 60	273	11.08 ± 8.11			175	98			132	141	
Gender				0.5179				0.2663				0.5393
	male	360	11.54 ± 9.46			224	136			183	177	
	female	130	10.93 ± 8.49			88	42			62	68	
Subsite				0.0004				0.0834				0.2659
	oral cavity	300	10.21±7.42			200	100			156	144	
	non-oral cavity	190	13.22±11.26			112	78			89	101	
^1^HPV status				0.0001				0.00003				0.0019
	HPV^+^	69	18.26±15.68			29	40			23	46	
	HPV^-^	407	10.18±7.11			277	130			218	189	
Smoking				0.9258				0.8155				0.5142
	ever/active	305	11.41±9.33			193	112			156	149	
	no	185	11.33±9.03			119	66			89	96	
Alcohol				0.5027				0.1837				0.0858
	ever/active	324	11.18±9.49			213	111			171	153	
	no	166	11.77±8.65			99	67			74	92	
Grade				0.056				0.8167				0.3813
	1	60	10.33±6.50			39	21			29	31	
	2	293	10.33±8.67			195	98			155	138	
	3	118	13.05±11.33			70	48			55	63	
^2^PNI				0.0177				0.0331				0.0830
	Yes	163	9.34±6.67			116	47			90	73	
	No	181	11.24±7.97			109	72			83	98	
^3^LVI				0.3676				0.4713				0.9545
	Yes	119	11.22±9.88			76	43			61	58	
	No	214	10.34±7.68			145	69			109	105	
^4^AJCC T				0.0015				0.0002				0.0012
	1+2	189	11.73±6.10			101	88			77	112	
	3+4	301	11.02±7.06			211	90			168	133	
AJCC N				0.2593				0.8960				0.2676
	0	222	10.90±7.22			140	82			104	118	
	N+	264	11.85±10.64			168	96			137	127	
Stage				0.6639				0.0873				0.0483
	1+2	106	11.72±7.36			60	46			44	62	
						252	132			201	183	

**^1^HPV**: human papillomavirus; **^2^PNI**: perineural invasion; **^3^LVI**: lymphovascular invasion; **^4^AJCC**: American Joint Committee of Cancer

**Table 2:** The associations of *ALDH2* mRNA level with clinical variables by anatomic subsite

		Oral Cavity			Non-Oral Cavity				
Characteristics		*n* = 300	*ALDH2* level Mean ± SD	*p*-value		*n* = 190	*ALDH2* level Mean ± SD	*p*-value	
**Age**				0.5119				0.7336	
	< 60	129	10.51±8.82			87	13.52±12.34		
	≥ 60	170	9.94±6.18			103	12.96±10.31		
**Gender**				0.8097				0.8042	
	male	201	10.14±8.07			159	13.31±10.74		
	female	99	10.36±5.93			31	12.76±13.80		
**^1^HPV status**				0.1057				0.0001	
	HPV+	18	12.92±16.32			136	10.60±8.11		
	HPV-	271	9.96±6.55			51	20.15±15.16		
**Grade**				0.7669				0.0351	
	1+2	236	10.19±7.22			117	11.82±10.18		
	3	60	10.51±8.34			58	15.68±13.33		
**Smoking**				0.8542				0.5763	
	ever/active	174	10.28±6.93			131	12.91±11.66		
	no	126	10.12±8.09			59	13.90±10.38		
**Alcohol**				0.4676				0.5896	
	ever/active	192	9.98±7.65			132	12.92±11.47		
	no	108	10.63±7.02			58	13.88±10.82		
**^2^AJCC T**				0.0017				0.0489	
	1+2	125	11.80±8.93			64	15.47±12.40		
	3+4	175	9.08±5.90			126	12.07±10.50		
**AJCC N**				0.1804				0.0173	
	0	142	10.86±6.86			80	10.97±7.85		
	N+	155	9.70±7.93			109	14.91±13.03		
**Stage**				0.1003				0.7053	
	1+2	76	11.42±6.68			30	12.50±8.92		
	3+4	224	9.80±7.63			160	13.35±11.66		
**^3^PNI**				0.1145				0.0392	
	Yes	131	9.40±7.02			32	9.08±5.04		
	No	103	10.94±7.82			78	13.28±10.89		
**^4^LVI**				0.4179				0.9350	
	Yes	69	10.57±9.48			50	12.12±10.43		
	No	153	9.69±6.38			61	11.96±10.13		

**^1^HPV**: human papillomavirus; **^2^AJCC**: American Joint Committee of Cancer; **^3^PNI**: perineural invasion; **^4^LVI**: lymphovascular invasion

**Table 3:** The associations of *ALDH2* mRNA level with clinical variables by HPV status

		^1^HPV^-^								HPV^+^					
Characteristics		*n* = 407	*ALDH*2 level, Mean ± SD	*p*-value		*ALDH2*-L	*ALDH2*-H *p*-value			*n* = 69	*ALDH*2 level Mean ± SD	*p*-value	*ALDH2*-L	*ALDH2*-H *p*-value	
**Age**				0.3502				0.1271				0.7408			0.7933
	< 60	167	9.78±6.99			97	70			44	19.18±16.91		14	30	
	≥ 60	240	10.45±7.19			121	119			25	16.65±13.40		9	16	
**Gender**				0.6042				0.2064				0.4802			1
	male	286	10.27±6.98			159	127			64	18.27±15.06		21	43	
	female	121	10.67±7.41			59	62			5	18.17±24.62		2	3	
**Subsite**				0.3921				0.6498				0.0096			0.0394
	oral cavity	271	9.96±6.55			143	128			18	12.92±16.32		10	8	
	non-oral cavity	136	10.60±8.11			75	61			51	20.15±15.16		13	38	
**Grade**				0.1547				0.5841				0.9			1
	1+2	312	9.95±6.24			170	142			30	18.74±18.59		10	20	
	3	86	11.19±9.75			44	42			30	17.97±14.11		11	19	
**Smoking**				0.7630				0.6197				0.7668			1
	ever/active	255	10.26±7.41			139	116			39	18.63±16.03		13	26	
	no	152	10.04±6.59			79	73			30	17.78±15.46		10	20	
**Alcohol**				0.0277				0.0338				0.6067			1
	ever/active	259	9.59±6.95			149	110			55	18.44±15.35		18	37	
	no	148	11.20±7.28			69	79			14	17.56±17.51		5	9	
**^2^AJCC T**				0.0341				0.0116				0.3375			0.4477
	1+2	144	11.18±6.94			65	79			38	20.08±17.05		11	27	
	3+4	263	9.62±7.15			153	110			31	16.04±13.76		12	19	
**AJCC N**				0.4397				0.0877				0.1586			0.4171
	0	195	10.49±6.50			95	100			22	14.12±11.96		9	13	
	N+	208	9.94±7.68			119	89			47	20.20±16.91		14	33	
**Stage**				0.2056				0.0371				0.6277			1
	1+2	89	11.02±6.09			39	50			14	15.81±12.72		5	9	
	3+4	318	9.94±7.36			179	139			55	18.89±16.39		18	37	

**^1^HPV**: human papillomavirus; **^2^AJCC**: American Joint Committee of Cancer

**Table 4:** The correlation of *ALDH2* level and the IC_50_ of systemic agents

Drug	*ALDH2* level	
	Correlation	*p*- value
**Docetaxel**	-0.214	0.241
**Paclitaxel**	-0.192	0.529
**Cisplatin**	-0.099	0.748
**5- fluorouracil**	-0.593	0.033*
**Methotrexate**	-0.055	0.429
**Cetuximab**	-0.005	0.493

## References

[1] Leemans CR, Snijders PJF, Brakenhoff RH (2018). The molecular landscape of head and neck cancer. Nat Rev Cancer.

[2] Cancer Genome Atlas Network (2015). Comprehensive genomic characterization of head and neck squamous cell carcinomas. Nature.

[3] Chen CH, Ferreira JC, Gross ER, Mochly-Rosen D (2014). Targeting aldehyde dehydrogenase 2: new therapeutic opportunities. Physiol Rev.

[4] Kimura M, Yokoyama A, Higuchi S (2019). Aldehyde dehydrogenase-2 as a therapeutic target. Expert Opin Ther Targets.

[5] Langevin F, Crossan GP, Rosado IV, Arends MJ, Patel KJ (2011). Fancd2 counteracts the toxic effects of naturally produced aldehydes in mice. Nature.

[6] Garaycoechea JI, Crossan GP, Langevin F, Mulderrig L, Louzada S, Yang F (2018). Alcohol and endogenous aldehydes damage chromosomes and mutate stem cells. Nature.

[7] Chen X, Legrand AJ, Cunniffe S, Hume S, Poletto M, Vaz B (2018). Interplay between base excision repair protein XRCC1 and ALDH2 predicts overall survival in lung and liver cancer patients. Cell Oncol (Dordr).

[8] Li K, Guo W, Li Z, Wang Y, Sun B, Xu D (2019). ALDH2 Repression Promotes Lung Tumor Progression via Accumulated Acetaldehyde and DNA Damage. Neoplasia.

[9] Ganesan M, Krutik VM, Makarov E, Mathews S, Kharbanda KK, Poluektova LY (2019). Acetaldehyde suppresses the display of HBV-MHC class I complexes on HBV-expressing hepatocytes. Am J Physiol Gastrointest Liver Physiol.

[10] Hu J, Yang L, Peng X, Mao M, Liu X, Song J (2022). ALDH2 Hampers Immune Escape in Liver Hepatocellular Carcinoma through ROS/Nrf2-mediated Autophagy. Inflammation.

[11] Zhang H, Xia Y, Wang F, Luo M, Yang K, Liang S (2021). Aldehyde Dehydrogenase 2 Mediates Alcohol-Induced Colorectal Cancer Immune Escape through Stabilizing PD-L1 Expression. AdvSci (Weinh).

[12] Hou G, Chen L, Liu G, Li L, Yang Y, Yan HX (2017). Aldehyde dehydrogenase-2 (ALDH2) opposes hepatocellular carcinoma progression by regulating AMP-activated protein kinase signaling in mice. Hepatology.

[13] Gao YH, Wu ZX, Xie LQ, Li CX, Mao YQ, Duan YT (2017). VHL deficiency augments anthracycline sensitivity of clear cell renal cell carcinomas by down-regulating ALDH2. Nat Commun.

[14] Wang NN, Wang LH, Li Y, Fu SY, Xue X, Jia LN (2018). Targeting ALDH2 with disulfiram/copper reverses the resistance of cancer cells to microtubule inhibitors. Exp Cell Res.

[15] Lefebvre JL, Chevalier D, Luboinski B, Kirkpatrick A, Collette L, Sahmoud T (1996). Larynx preservation in pyriform sinus cancer: preliminary results of a European Organization for Research and Treatment of Cancer phase III trial. EORTC Head and Neck Cancer Cooperative Group. J Natl Cancer Inst.

[16] Lefebvre JL, Andry G, Chevalier D, Luboinski B, Collette L, Traissac L (2012). Laryngeal preservation with induction chemotherapy for hypopharyngeal squamous cell carcinoma: 10-year results of EORTC trial 24891. Ann Oncol.

[17] Abdelmeguid AS, Silver NL, Boonsripitayanon M, Glisson BS, Ferrarotto R, Gunn GB (2021). Role of induction chemotherapy for oral cavity squamous cell carcinoma. Cancer.

[18] Patil VM, Prabhash K, Noronha V, Joshi A, Muddu V, Dhumal S (2014). Neoadjuvant chemotherapy followed by surgery in very locally advanced technically unresectable oral cavity cancers. Oral Oncol.

[19] Gangopadhyay A, Bhatt S, Nandy K, Rai S, Rathod P, Puj KS (2021). Survival Impact of Surgical Resection in Locally Advanced T4b Oral Squamous Cell Carcinoma. Laryngoscope.

[20] Hsieh MY, Chen G, Chang DC, Chien SY, Chen MK (2018). The Impact of Metronomic Adjuvant Chemotherapy in Patients with Advanced Oral Cancer. Ann Surg Oncol.

[21] Fucikova J, Kepp O, Kasikova L, Petroni G, Yamazaki T, Liu P (2020). Detection of immunogenic cell death and its relevance for cancer therapy. Cell Death Dis.

[22] Mizumoto A, Ohashi S, Hirohashi K, Amanuma Y, Matsuda T, Muto M (2017). Molecular Mechanisms of Acetaldehyde-Mediated Carcinogenesis in Squamous Epithelium. Int J Mol Sci.

[23] Alcón P, Shakeel S, Chen ZA, Rappsilber J, Patel KJ, Passmore LA (2020). FANCD2-FANCI is a clamp stabilized on DNA by monoubiquitination of FANCD2 during DNA repair. Nat Struct Mol Biol.

[24] Almeida LO, Goto RN, Pestana CR, Uyemura SA, Gutkind S, Curti C (2012). SET overexpression decreases cell detoxification efficiency: ALDH2 and GSTP1 are downregulated, DDR is impaired and DNA damage accumulates. FEBS J.

[25] Zhong S, Li L, Zhang YL, Zhang L, Lu J, Guo S (2019). Acetaldehyde dehydrogenase 2 interactions with LDLR and AMPK regulate foam cell formation. J Clin Invest.

[26] Tan X, Chen YF, Zou SY, Wang WJ, Zhang NN, Sun ZY (2023). ALDH2 attenuates ischemia and reperfusion injury through regulation of mitochondrial fusion and fission by PI3K/AKT/mTOR pathway in diabetic cardiomyopathy. Free Radic Biol Med.

[27] Guo Q, Zhang T, Gong Y, Tao Y, Gao Y, Wang Y (2022). Aldehyde dehydrogenase 6 family member A1 negatively regulates cell growth and to cisplatin sensitivity in bladder cancer. Mol Carcinog.

[28] Li X, Wang N, Wu Y, Liu Y, Wang R (2022). ALDH6A1 weakens the progression of colon cancer via modulating the RAS/RAF/MEK/ERK pathway in cancer cell lines. Gene.

[29] Andreuzzi E, Fejza A, Polano M, Poletto E, Camicia L, Carobolante G (2022). Colorectal cancer development is affected by the ECM molecule EMILIN-2 hinging on macrophage polarization via the TLR-4/MyD88 pathway. J Exp Clin Cancer Res.

[30] Kratochvill F, Neale G, Haverkamp JM, Van de Velde LA, Smith AM, Kawauchi D (2015). TNF Counterbalances the Emergence of M2 Tumor Macrophages. Cell Rep.

[31] Yue S, Rao J, Zhu J, Busuttil RW, Kupiec-Weglinski JW, Lu L (2014). Myeloid PTEN deficiency protects livers from ischemia reperfusion injury by facilitating M2 macrophage differentiation. J Immunol.

[32] Kumar AT, Knops A, Swendseid B, Martinez-Outschoom U, Harshyne L, Philp N (2019). Prognostic Significance of Tumor-Associated Macrophage Content in Head and Neck Squamous Cell Carcinoma: A Meta-Analysis. Front Oncol.

[33] Helmink BA, Reddy SM, Gao J, Zhang S, Basar R, Thakur R (2020). B cells and tertiary lymphoid structures promote immunotherapy response. Nature.

[34] Nielsen JS, Sahota RA, Milne K, Kost SE, Nesslinger NJ, Watson PH (2012). CD20+ tumor-infiltrating lymphocytes have an atypical CD27- memory phenotype and together with CD8+ T cells promote favorable prognosis in ovarian cancer. Clin Cancer Res.

[35] Iglesia MD, Parker JS, Hoadley KA, Serody JS, Perou CM, Vincent BG (2016). Genomic Analysis of Immune Cell Infiltrates Across 11 Tumor Types. J Natl Cancer Inst.

[36] Sautès-Fridman C, Verneau J, Sun CM, Moreira M, Chen TW, Meylan M (2020). Tertiary Lymphoid Structures and B cells: Clinical impact and therapeutic modulation in cancer. Semin Immunol.

[37] Ruffin AT, Cillo AR, Tabib T, Liu A, Onkar S, Kunning SR (2021). B cell signatures and tertiary lymphoid structures contribute to outcome in head and neck squamous cell carcinoma. Nat Commun.

[38] Lu N, Li Y, Zhang Z, Xing J, Sun Y, Yao S (2018). Human Semaphorin-4A drives Th2 responses by binding to receptor ILT-4. Nat Commun.

