# Supplementary material for: Analysis of Aldehyde Dehydrogenase 2 as a Prognostic Marker Associated with Immune Cell infiltration and Chemotherapy Efficacy in Head and Neck Squamous Cell Carcinoma
Source: J Cancer. 2023 Jun 12;14(10):1689–706. doi: 10.7150/jca.85098 (PMC10355205; doi:10.7150/jca.85098)
Supplement: Supplementary file 1 — Supplementary figures and tables. [file jcav14p1689s1.pdf]

Supplementary documentation

Supplementary Tables

Table S1. The Top 30 Mutated Genes in TCGA-HNSC cohort (*n* = 515)

Gene	Mutation Significance (Q-value)	Number of Mutation	Frequency
TP53	436	357	69.30%
FAT1	142	111	21.60%
CDKN2A	112	105	20.40%
PIK3CA	94	90	17.50%
NOTCH1	97	88	17.10%
LRP1B	111	85	16.50%
PCLO	92	79	15.30%
KMT2D	88	77	15.00%
NSD1	76	60	11.70%
CASP8	63	55	10.70%
RELN	53	48	9.30%
FAT4	46	41	8.00%
KMT2C	38	37	7.20%
EP300	37	37	7.20%
FBXW7	35	33	6.40%
HRAS	34	31	6.00%
CREBBP	35	30	5.80%
RNF213	32	30	5.80%
AJUBA	32	29	5.60%
MGAM	34	29	5.60%
PRKDC	31	29	5.60%
LRRK2	35	29	5.60%
NFE2L2	29	28	5.40%
SPEN	30	27	5.20%
PTPRD	29	27	5.20%
GRM3	30	26	5.00%
ROS1	28	26	5.00%
KMT2A	25	25	4.90%
EPHA5	26	25	4.90%
TGFBR2	26	24	4.70%

Table S2. The correlation of immunohistochemistry expression levels of ALDH2 with clinical variables (n = 101)

**Characteristics**		**Number**	**ALDH2, Mean ± [1]SD**	***p*-value**
**Age**				0.3816
	< 60	61	22.69±20.75	
	≥ 60	40	27.15±23.16	
**Gender**				0.5958
	male	92	24.77±21.97	
	female	9	21.34±19.96	
**Subsite**				0.0027
	oral cavity	23	14.28±16.51	
	larynx	72	27.11±21.61	
				0.0037
**Grade**	1	25	16.09±17.89	
	2/3	72	28.52±22.16	
				0.1039
[2]**AJCC T**	1+2	42	20.79±20.53	
	3+4	59	27.07±22.35	
				0.6652
**AJCC N**	0	76	24.85±21.44	
	N+	25	23.28±23.00	

[1]**SD**: standard deviation; [2]**AJCC**: American Joint Committee on Cancer

Supplementary Figure Legends

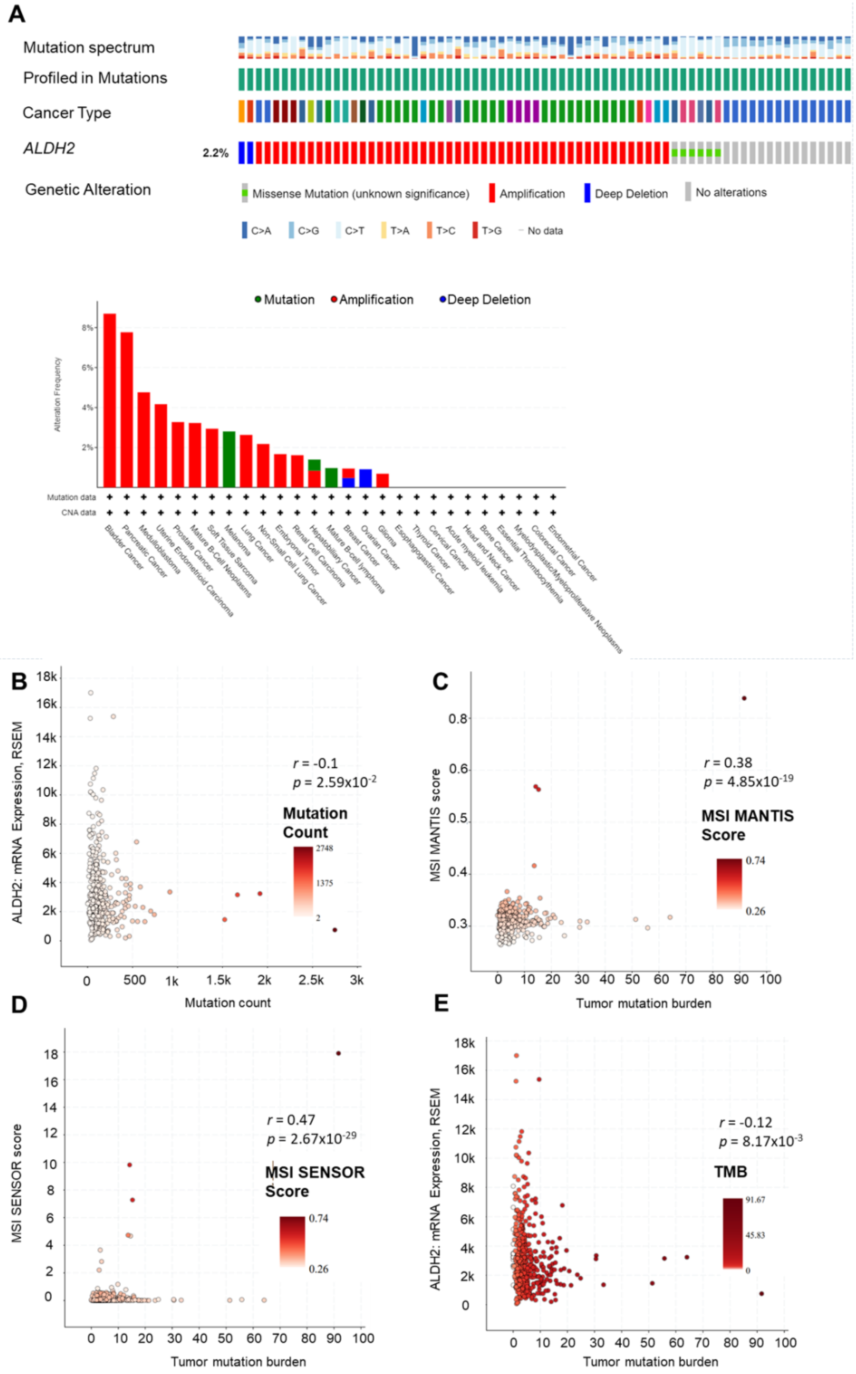

Figure S1. The genetic analysis of *ALDH2* in cancer. (A) The cBioPortal presents the pattern and frequency of genetic alterations of *ALDH2* in pan-cancer, as well as the proportion of *ALDH2* copy number alterations in different cancer types. (B) The relationship between *ALDH2* levels and mutation count. (C, D) The associations of tumor mutation burden (TMB) with microsatellite instability within the TCGA-HNSC cohort. (E) The correlation between *ALDH2* transcripts and TMB in 507 HNSC patients.

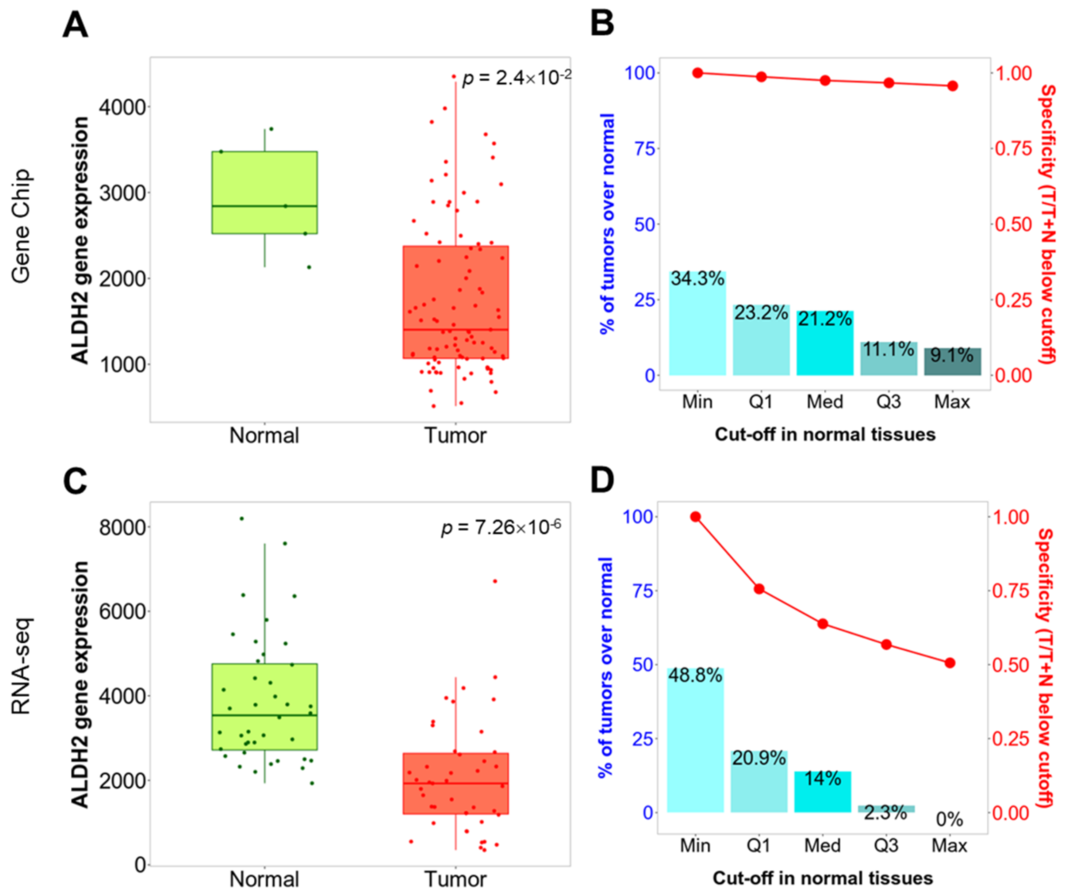

Figure S2. The differential expression of *ALDH2* in tumor and normal tissues. (A, C) The box plots in TNMplot reveal the *ALDH2* transcripts in normal- and tumor tissues on a gene chip and RNA-seq data. (B, D) The bar plot shows the ratio of tumor *ALDH2* levels to normal tissue *ALDH2* levels at different cut-off values.

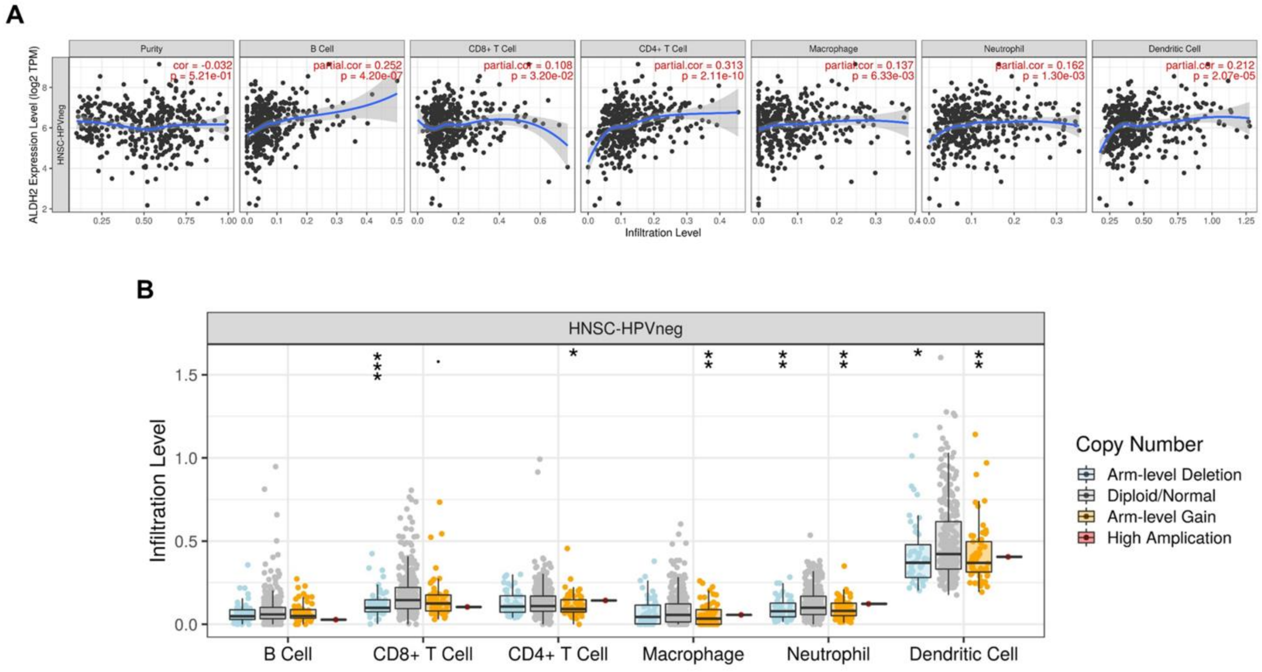

Figure S3. The association between *ALDH2* level and tumor immune infiltration in HPV-unrelated HNSC TIMER presents. (A) The *ALDH2* levels within six infiltrated immune cells, and (B) the effect of *ALDH2* copy number variation on immune cell infiltration levels in head and neck cancer, with asterisks indicating significant associations.

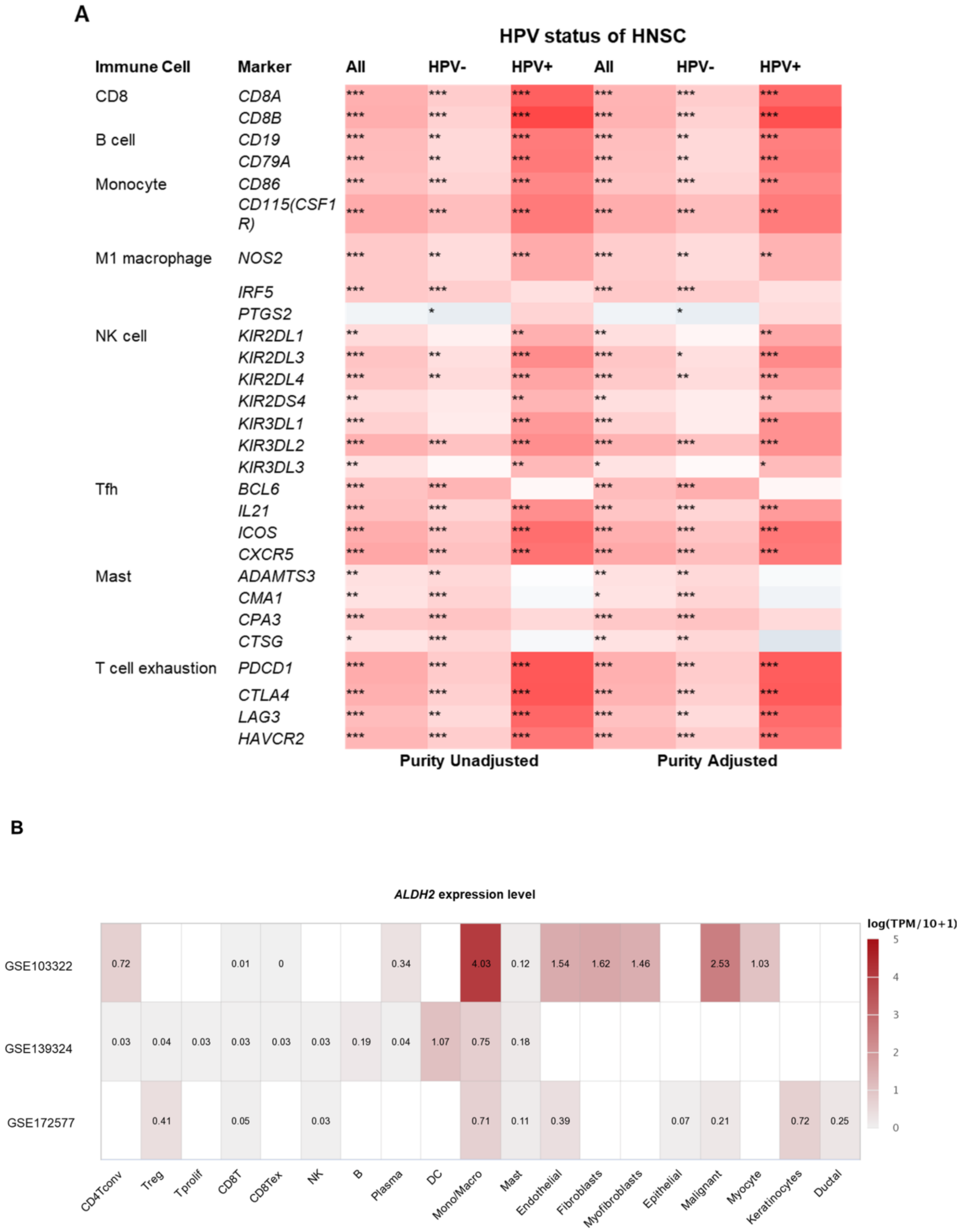

Figure S4. The immunological features of *ALDH2* level in HNSC. (A) The heatmap presents the adjusted and non-adjusted correlations of gene markers of the tumor-infiltrating immune cells and *ALDH2* levels in HNSC by HPV statuses. Statistical significance: *$P < 0.05$, **$P < 0.01$, ***$P < 0.001$. (B) The TISCH database revealed the *ALDH2* levels in various cell types within the HNSC tumor microenvironment across three GEO cohorts.

A

cancer	MYD88	PTEN	TNFRSF1A
HNSC (n=522)	0.139	0.176	0.054
HNSC-HPV- (n=422)	0.142	0.219	0.136
HNSC-HPV+ (n=98)	0.194	0.056	-0.142

B

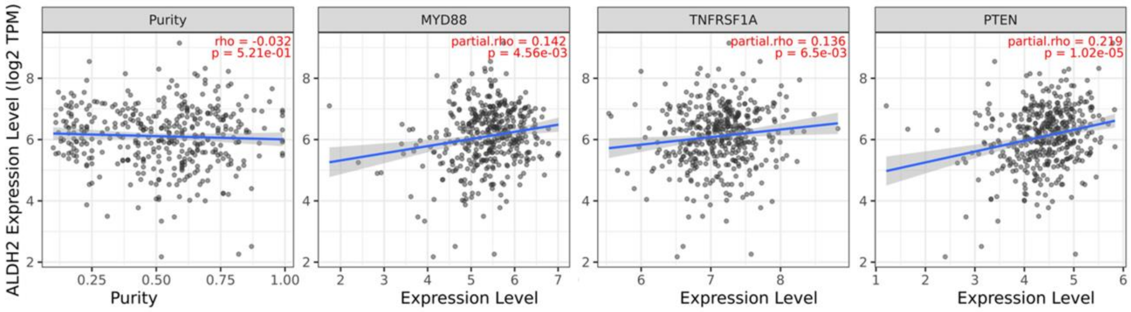

Figure S5. The correlation between *ALDH2* levels with molecules involved in macrophage polarization. (A) The TIMER 2.0 data port demonstrated significant correlations for *Myd88*, *PTEN*, and *TNFRSF1A* in HPV-unrelated HNSC but not in HPV-related HNSC. (B) The adjusted correlation coefficients for *Myd88*, *TNFRSF1A*, and *PTEN* in HPV- group.
